# The cervical microbiota of Hispanics living in Puerto Rico is nonoptimal regardless of HPV status

**DOI:** 10.1128/msystems.00357-23

**Published:** 2023-08-03

**Authors:** Daniela Vargas-Robles, Josefina Romaguera, Ian Alvarado-Velez, Eduardo Tosado-Rodríguez, Anelisse Dominicci-Maura, Maria Sanchez, Kara J. Wiggin, Magaly Martinez-Ferrer, Jack A. Gilbert, Larry J. Forney, Filipa Godoy-Vitorino

**Affiliations:** 1 Department of Microbiology and Medical Zoology, University of Puerto Rico School of Medicine, Medical Sciences Campus, San Juan, Puerto Rico; 2 Department of Obstetrics and Gynecology, University of Puerto Rico School of Medicine, Medical Sciences Campus, San Juan, Puerto Rico; 3 University of Puerto Rico Comprehensive Cancer Center, San Juan, Puerto Rico; 4 Department of Pediatrics, University of California San Diego, La Jolla, California, USA; 5 Scripps Institution of Oceanography, University of California San Diego, La Jolla, California, USA; 6 Department of Biological Sciences, University of Idaho, Moscow, Idaho, USA; Quadram Institute Bioscience, Norwich, Norfolk, United Kingdom

**Keywords:** cervicovaginal microbiota, 16S rRNA genes, human papilloma viruses (HPV), cervical lesions

## Abstract

The cervicovaginal microbiota is influenced by host physiology, immunology, lifestyle, and ethnicity. We hypothesized that there would be differences in the cervicovaginal microbiota among pregnant, nonpregnant, and menopausal women living in Puerto Rico (PR) with and without human papillomavirus (HPV) infection and cervical cancer. We specifically wanted to determine if the microbiota is associated with variations in cervical cytology. A total of 294 women, including reproductive-age nonpregnant (*N* = 196), pregnant (*N* = 37), and menopausal (*N* = 61) women, were enrolled. The cervicovaginal bacteria were characterized by 16S rRNA amplicon sequencing, the HPV was genotyped with SPF10-LiPA, and cervical cytology was quantified. High-risk HPV (HR-HPV, 67.3%) was prevalent, including genotypes not covered by the 9vt HPV vaccine. Cervical lesions (34%) were also common. The cervical microbiota was dominated by *Lactobacillus iners*. Pregnant women in the second and third trimesters exhibited a decrease in diversity and abundance of microbes associated with bacterial vaginosis. Women in menopause had greater alpha diversity, a greater proportion of facultative and strictly anaerobic bacteria, and higher cervicovaginal pH than premenopausal women. Cervical lesions were associated with greater alpha diversity. However, no significant associations between the microbiota and HPV infection (HR or LR-HPV types) were found. The cervicovaginal microbiota of women living in Puerto Rican were either dominated by *L. iners* or diverse microbial communities regardless of a woman’s physiological stage. We postulate that the microbiota and the high prevalence of HR-HPV increase the risk of cervical lesions among women living in PR.

**IMPORTANCE:**

In the enclosed manuscript, we provide the first in-depth characterization of the cervicovaginal microbiota of Hispanic women living in Puerto Rico (PR), using a 16S rRNA approach, and include women of different physiological stages. Surprisingly we found that high-risk HPV was ubiquitous with a prevalence of 67.3%, including types not covered by the 9vt HPV vaccine. We also found highly diverse microbial communities across women groups—with a reduction in pregnant women, but dominated by nonoptimal *Lactobacillus iners*. Additionally, we found vaginosis-associated bacteria as *Dialister* spp., *Gardnerella* spp., *Clostridium*, or *Prevotella* among most women. We believe this is a relevant and timely article expanding knowledge on the cervicovaginal microbiome of PR women, where we postulate that these highly diverse communities are conducive to cervical disease.

## INTRODUCTION

Cervical cancer remains the fourth most common cancer in women worldwide, affecting 13.1 of every 100,000 women (1). Cervical cancer incidence and mortality rates in Latin America and the Caribbean are around 30% to 40% higher than in nonHispanic white women (2). Hispanic women living in Puerto Rico (PR) have a higher incidence of cervicovaginal cancer (12.9 vs 7.2 per 100,000), as well as a higher death rate when compared to women in the U.S. mainland (2.2 vs 2.0 per 100,000) (2, 3). A recent study confirmed that women living in PR have the highest age-adjusted incidence of cervical cancer in the U.S., with an increasing incidence from 2001 to 2017 from 9.2 to 13 per 100,000 person-years (4).

Cervical carcinogenesis is a complex process influenced by the host and the microbiota (5). One key element of cervical cancer development and an etiological agent of the disease is the persistence of human papillomavirus (HPV) (6, 7). HPV, particularly high-risk HPV (HR-HPV) genotypes, increases the risk of cervical neoplasia (8). These cervical abnormalities are detected through cytology, which provides a way to identify different squamous cell changes known as atypical squamous cells of undetermined significance (ASCUS), low-grade or high-grade squamous intraepithelial lesions (LGSIL and HGSIL, respectively) (9).

Recent evidence indicates that the cervicovaginal environment, including the microbiota, may play a role in viral persistence and the progression of epithelial lesions that lead to carcinogenesis (10). Differential activation of the mucosal immune system by varying cervicovaginal microbiota composition may play a role in lesion development (11). The composition of the cervicovaginal microbiota is influenced by ethnicity, age, and lifestyle. For example, the cervicovaginal microbiota of White women is dominated by *Lactobacillus crispatus*, while Hispanic and Black women have a higher prevalence of non*Lactobacillus* genera such as *Dialister, Gardnerella, Clostridium, or Prevotella* (12). Women living in PR (mainly Puerto Ricans) are Hispanics with a genetic admixture of European, Amerindian, and African ancestry. This population, however, differs from U.S. and Mexican Hispanics due to a greater contribution of African genetic ancestry (<6.2% vs 16–32%) (13 - 15). The cervicovaginal microbiota of women living in PR reproductive-age women is dominated by *L. iners* (16). In addition to ancestry, factors such as physiological, lifestyle, and hormonal changes throughout a woman’s life can significantly influence the vaginal microbiome (17); however, the cervicovaginal microbiota of pregnant and menopausal Puerto Rican women has not yet been characterized.

We posited that the cervicovaginal microbial diversity of Hispanic women residing in PR would vary with pregnancy, menopause, and HPV infection state. We found that *L. iners* and other diverse microbial cervicovaginal taxa were commonly found in Puerto Rican women regardless of their physiological stage, and that these microbes, in combination with HR-HPV, may promote a favorable environment for cervical lesion development.

## RESULTS

### Population description

A total of 333 samples from 294 women were included in the analyses (Table S1). This study cohort included three groups of women: reproductive-age non-pregnant (*N* = 196), pregnant (*N* = 37), and menopausal (*N* = 61) that differed in terms of the severity of cervical lesions and HPV infection state (Table 1).

**TABLE 1:** Study population description by women’s group

Variables	Nonpregnant (*N* = 196)	Pregnant (*N* = 37)	Menopause (*N* = 61)	Total (*N* = 294)	*P* value^*a*^
Age					*<1e-16*
Mean (SD)	36.0 (8.71)	28.3 (4.69)	52.1 (7.25)	38.4 (11.00)	
Median [min, max]	35.5 [21.0, 54.0]	28.0 [21.0, 43.0]	54.0 [28.0, 60.0]	37.0 [21.0, 60.0]	
BMI category					*0.003*
Normal	79 (40.3%)	7 (18.9%)	18 (29.5%)	104 (35.4%)	
Underweight	4 (2.0%)	1 (2.7%)	0 (0%)	5 (1.7%)	
Overweight	39 (19.9%)	12 (32.4%)	26 (42.6%)	77 (26.2%)	
Obese	73 (37.2%)	17 (45.9%)	16 (26.2%)	106 (36.1%)	
Missing	1 (0.5%)	0 (0%)	1 (1.6%)	2 (0.7%)	
Vaginal pH					*1e-8*
Mean (SD)	5.40 (0.540)	5.05 (0.522)	5.67 (0.469)	5.42 (0.549)	
Cervical lesions^*b*^	67 (34.2%)	20 (54.1%)	13 (21.3%)	100 (34.0%)	*0.001*
Type of cervical lesion^*c*^					*0.007*
HGSIL	33 (16.8%)	12 (32.4%)	6 (9.8%)	51 (17.3%)	
LGSIL	34 (17.3%)	8 (21.6%)	7 (11.5%)	49 (16.7%)	
NILM	114 (58.2%)	13 (35.1%)	45 (73.8%)	172 (58.5%)	
Missing	15 (7.7%)	4 (10.8%)	3 (4.9%)	22 (7.5%)	
HPV status	151 (77.0%)	27 (73.0%)	42 (68.9%)	220 (74.8%)	*0.444*
HPV type^*d*^					*0.300*
Only HR-HPV	84 (42.9%)	21 (56.8%)	28 (45.9%)	133 (45.2%)	
Only LR-HPV	17 (8.7%)	1 (2.7%)	2 (3.3%)	20 (6.8%)	
Both	49 (25.0%)	5 (13.5%)	11 (18.0%)	65 (22.1%)	
Negative	44 (22.4%)	10 (27.0%)	18 (29.5%)	72 (24.5%)	
Missing	2 (1.0%)	0 (0%)	2 (3.3%)	4 (1.4%)	
HPV type					*0.441*
HR-HPV type	133 (67.9%)	26 (70.3%)	39 (63.9%)	198 (67.3%)	
Only LR-HPV types	17 (8.7%)	1 (2.7%)	2 (3.3%)	20 (6.8%)	
Negative	44 (22.4%)	10 (27.0%)	18 (29.5%)	72 (24.5%)	
Missing	2 (1.0%)	0 (0%)	2 (3.3%)	4 (1.4%)	
Number of HR-HPV genotypes					*0.654*
Mean (SD)	1.77 (0.953)	1.54 (0.706)	1.95 (1.39)	1.77 (1.03)	
Number of LR-HPV genotypes					*0.159*
Mean (SD)	1.24 (0.432)	1.00 (0)	1.07 (0.267)	1.20 (0.401)	
HPV vaccine	40 (20.4%)	5 (13.5%)	3 (4.9%)	48 (16.3%)	*0.006*
Alcohol drinking	99 (50.5%)	1 (2.7%)	28 (45.9%)	128 (43.5%)	*1e-8*
Tobacco smoking	27 (13.8%)	4 (10.8%)	4 (6.6%)	35 (11.9%)	*0.311*
Heterosexual preference	191 (97.4%)	36 (97.3%)	61 (100%)	288 (98.0%)	*0.561*
Number of sexual partners					*1e-8*
Mean (SD)	4.94 (4.15)	4.82 (4.72)	2.36 (1.39)	4.38 (3.95)	
					
Antibiotic intake last 2 months	12 (6.1%)	3 (8.1%)	6 (9.8%)	21 (7.1%)	*0.549*
Pregnancy trimester					*1.000*
First	-	8 (21.6%)	-	8 (2.7%)	
Second	-	20 (54.1%)	-	20 (6.8%)	
Third	-	9 (24.3%)	-	9 (3.1%)	

^*a*^ Fisher’s exact test and Kruskal–Wallis test for categorical and continous variables, respectively.

^*b*^ Positive samples include the HGSIL or LGSIL.

^*c*^ Samples diagnosed as ASCUS and HPV positive were considered LGSIL.

^*d*^ HR-HPV: high-risk HPV; LR-HPV: low-risk HPV.

We first compared the demographic and health characteristics of women in these three groups. The median age was 35.5 for nonpregnant, 28 for pregnant, and 54 for menopause. The body mass index (BMI) for the nonpregnant women was mostly normal (40.3% of women), while most menopausal women were overweight (42.6%, *P* < 0.003). Pregnant women showed the highest prevalence of high-grade squamous intraepithelial lesion (HGSIL; 32.4%), while menopausal women had the lowest (9.8%). HPV infections were present in most subjects (74.8%) with no differences among groups of women. Only 16.3% of women were HPV vaccinated, mainly in the nonpregnant group (20.4%). Regular alcohol drinking occurred in 43.7% of the women, mostly in nonpregnant and menopausal women, and only one pregnant woman reported drinking regularly (2.7%). Tobacco smoking was less common (11.9%) and similarly distributed among groups. Most subjects were self-declared heterosexuals (98.0%) and reported a sexual history of three sexual partners, which was higher in nonpregnant women (median: four sexual partners). The use of antibiotics for the last two months was reported only in 7.1% of women, and 1.0% did not answer. These women were retained in the study since they were uniformly distributed among women’s groups. However, this variable was always included in the models as covariable. Pregnant women were mostly in their second pregnancy trimester (54.1%), followed by third (24.3%) and first (21.6%, Table 1).

### Cohort description according to cervical lesion and HPV status

We compared the prevalence of HPV infection and cervical lesions across women’s groups. We found an overall prevalence for cervical lesions of 34.0% (100/294) (Table 1), with 17.3% of HGSIL and 16.7% of low-grade squamous intraepithelial lesion (LGSIL). For any HPV genotype infection (HPV status), we found an overall prevalence of 74.8% (220/294), where 67.3% (198/294) of patients were HR-HPV positive. We also reported the prevalence of infections with only HR-HPV (45.2%, 133/294), with only LR-HPV (6.8%, 20/294) and mixed infections (both HR and LR-HPV types at the same time, 22.1%, 65/294, Table 1). Mixed infections were higher in LGSIL (30.6%) than in HGSIL (9.8%, *P* = 0.031, data not shown). No differences in HGSIL prevalence were observed comparing patients with single HPV 16 infection than when HPV 16 was accompanied by another HPV genotype (*P* > 0.050). As expected, HR-HPV prevalence was higher in HGSIL (76.5%) compared to LGSIL and negative cervical lesions (*Padj* < 1e-4, Table 2). HGSIL samples showed mostly infections with HR-HPV 16 (37.8%), HPV-HR 52 (28.9%), and HPV-HR 51 genotypes (24.4%) (Table 3). Interestingly, there were no cases of HGSIL observed, and only one sample with LGSIL was found to be related to HR-HPV-18 infections (Table 3). In terms of the number of HPV types, women had a median of 1 (mean of 1.8, max: 6) HR-HPV genotype and 1 (mean of 1.2, max: 2) LR-HPV genotype, with no difference among women groups (Table 1) or between lesion types (HR-HPV, *P* = 0.195; LR-HPV, *P* = 0.857; Kruskal–Wallis test, Table 2). The most prevalent HR-HPV genotypes were 51 (31.4%), 16 (20.9%), 33 (16.8%), 52 (14.5%), and 56 (14.5%), with similar prevalence among women’s groups (*P >* 0.246). Only HR-HPVs 59 and 31 were marginally more prevalent in menopausal women (11.9% and 9.5%, respectively, *P <* 0.024, *Padj >* 0.173, Table S2). Among the LR-HPV, genotypes 53 (22.7%), 74 (10.0%), and 44 (4.1%) were the most prevalent and not different among women groups.

**TABLE 2:** HPV infection prevalence, HPV number of genotypes, and community state types (CSTs) among women with differential cervical lesion statuses

Cervical lesion type^*a*^					
	NILM	LGSIL	HGSIL	TOTAL	
Variables	(*N* = 172)	(*N* = 49)	(*N* = 51)	(*N* = 294)	*P value^b^*
HPV status					*6.2e-5*
Positive	114 (66.3%)	45 (91.8%)	45 (88.2%)	220 (74.8%)	*Padj = 1e-4*
Negative	56 (32.6%)	4 (8.2%)	6 (11.8%)	72 (24.5%)	
Unknown	2 (1.2%)	0 (0%)	0 (0%)	2 (0.7%)	
HPV type^*c*^					*8.7e-5*
Any with HR-HPV type	104 (60.5%)	38 (77.6%)	44 (86.3%)	198 (67.3%)	*Padj = 1e-4*
Only LR-HPV	10 (5.8%)	6 (12.2%)	1 (2.0%)	20 (6.8%)	
Negative	56 (32.6%)	4 (8.2%)	6 (11.8%)	72 (24.5%)	
Missing	2 (1.2%)	1 (2.0%)	0 (0%)	4 (1.4%)	
HPV type					*2.8e-6*
Only HR-HPV	66 (38.4%)	23 (46.9%)	39 (76.5%)	133 (45.2%)	*Padj = 3e-5*
Only LR-HPV	10 (5.8%)	6 (12.2%)	1 (2.0%)	20 (6.8%)	
Mixed HPV infection	38 (22.1%)	15 (30.6%)	5 (9.8%)	65 (22.1%)	
Negative	56 (32.6%)	4 (8.2%)	6 (11.8%)	72 (24.5%)	
Missing	2 (1.2%)	1 (2.0%)	0 (0%)	4 (1.4%)	
Number of HR-HPV genotypes					*0.195*
Mean (SD)	1.87 (1.12)	1.84 (1.05)	1.52 (0.792)	1.77 (1.03)	
Median [Min, Max]	2.00 [1.00, 6.00]	2.00 [1.00, 5.00]	1.00 [1.00, 4.00]	1.00 [1.00, 6.00]	
Missing	68 (39.5%)	11 (22.4%)	7 (13.7%)	96 (32.7%)	
Number of LR-HPV genotypes					*0.857*
Mean (SD)	1.18 (0.391)	1.24 (0.436)	1.17 (0.408)	1.20 (0.401)	
Median [Min, Max]	1.00 [1.00, 2.00]	1.00 [1.00, 2.00]	1.00 [1.00, 2.00]	1.00 [1.00, 2.00]	
Missing	123 (71.5%)	28 (57.1%)	45 (88.2%)	208 (70.7%)	
Vaginal pH					*0.772*
Mean (SD)	5.44 (0.516)	5.35 (0.485)	5.46 (0.689)	5.42 (0.549)	
Median [Min, Max]	5.50 [4.00, 7.00]	5.25 [4.00, 6.00]	5.00 [4.00, 7.00]	5.50 [4.00, 7.00]	
Unknown	72 (41.9%)	15 (30.6%)	17 (33.3%)	110 (37.4%)	
CST^*d*^ I	36 (20.9%)	9 (18.4%)	6 (11.8%)	57 (19.4%)	*0.363*
I-A	25 (14.5%)	9 (18.4%)	4 (7.8%)	43 (14.6%)	*0.283*
I-B	11 (6.4%)	0 (0%)	2 (3.9%)	14 (4.8%)	*0.208*
II	3 (1.7%)	3 (6.1%)	1 (2.0%)	8 (2.7%)	*0.230*
III	66 (38.4%)	19 (38.8%)	25 (49.0%)	116 (39.5%)	*0.397*
III-A	51 (29.7%)	12 (24.5%)	17 (33.3%)	84 (28.6%)	*0.633*
III-B	15 (8.7%)	7 (14.3%)	8 (15.7%)	32 (10.9%)	*0.250*
IV	63 (36.6%)	15 (30.6%)	18 (35.3%)	104 (35.4%)	*0.771*
IV-A	1 (0.6%)	0 (0%)	0 (0%)	1 (0.3%)	*1.000*
IV-B	45 (26.2%)	11 (22.4%)	15 (29.4%)	79 (26.9%)	*1.000*
IV-C	17 (9.9%)	4 (8.2%)	3 (5.9%)	24 (8.2%)	*0.748*
IV-C0	6 (3.5%)	2 (4.1%)	2 (3.9%)	10 (3.4%)	*1.000*
IV-C1	7 (4.1%)	2 (4.1%)	1 (2.0%)	10 (3.4%)	*0.810*
IV-C2	3 (1.7%)	0 (0%)	0 (0%)	3 (1.0%)	*1.000*
IV-C3	1 (0.6%)	0 (0%)	0 (0%)	1 (0.3%)	*1.000*
V	4 (2.3%)	3 (6.1%)	1 (2.0%)	9 (3.1%)	*0.291*

^*a*^ Samples diagnosed as ASCUS and HPV positive were considered LGSIL.

^*b*^ Fisher’s exact test. *P* value adjustment with "False Discovery Rate" method.

^*c*^ HR-HPV: high-risk HPV, LR-HPV: low-risk HPV.

^*d*^ Fisher’s exact test was calculated by setting a particular CST as positive and considering the rest of the samples that do not belong to that CST as negative.

**TABLE 3:** Prevalence of HPV genotypes per cervical lesion groups including all women

	HGSIL	LGSIL	NILM	TOTAL	
HPV types^*b*^	(***N*** = 45)	(*N* = 45)	(*N* = 114)	(*N* = 220)	*P value^a^*
HR51	11 (24.4%)	13 (28.9%)	42 (36.8%)	69 (31.4%)	*0.301*
HR16	17 (37.8%)	7 (15.6%)	19 (16.7%)	46 (20.9%)	*0.012 (Padj = 0.092)*
HR33	6 (13.3%)	7 (15.6%)	23 (20.2%)	37 (16.8%)	*0.580*
HR52	13 (28.9%)	3 (6.7%)	14 (12.3%)	32 (14.5%)	*0.009 (Padj = 0.092)*
HR56	4 (8.9%)	10 (22.2%)	16 (14.0%)	32 (14.5%)	*0.193*
HR66	0 (0%)	5 (11.1%)	19 (16.7%)	29 (13.2%)	*0.005 (Padj = 0.092)*
HR45	5 (11.1%)	3 (6.7%)	7 (6.1%)	16 (7.3%)	*0.580*
HR35	5 (11.1%)	2 (4.4%)	7 (6.1%)	15 (6.8%)	*0.519*
HR68	0 (0%)	5 (11.1%)	10 (8.8%)	15 (6.8%)	*0.055*
HR39	1 (2.2%)	2 (4.4%)	8 (7.0%)	12 (5.5%)	*0.593*
HR58	2 (4.4%)	4 (8.9%)	4 (3.5%)	11 (5.0%)	*0.408*
HR59	0 (0%)	0 (0%)	8 (7.0%)	8 (3.6%)	*0.044 (Padj = 0.202)*
HR31	0 (0%)	2 (4.4%)	3 (2.6%)	5 (2.3%)	*0.407*
HR18	0 (0%)	1 (2.2%)	1 (0.9%)	2 (0.9%)	*0.689*
LR53	4 (8.9%)	12 (26.7%)	32 (28.1%)	50 (22.7%)	*0.023 (Padj = 0.132)*
LR74	3 (6.7%)	6 (13.3%)	11 (9.6%)	22 (10.0%)	*0.592*
LR44	0 (0%)	4 (8.9%)	4 (3.5%)	9 (4.1%)	*0.105*
LR6	0 (0%)	1 (2.2%)	2 (1.8%)	8 (3.6%)	*1.000*
LR70	0 (0%)	1 (2.2%)	4 (3.5%)	6 (2.7%)	*0.826*
LR54	0 (0%)	1 (2.2%)	2 (1.8%)	4 (1.8%)	*1.000*
LR40	0 (0%)	1 (2.2%)	1 (0.9%)	2 (0.9%)	*0.689*
LR19	0 (0%)	0 (0%)	1 (0.9%)	1 (0.5%)	*1.000*
LR43	0 (0%)	0 (0%)	1 (0.9%)	1 (0.5%)	*1.000*

^*a*^ Fisher’s exact test among total prevalence per cervical lesion group. *P* value adjustment with "False Discovery Rate" method.

^*b*^ HR = high risk HPV types, LR = low risk HPV types.

The cervicovaginal pH of nonpregnant women had a median of 5.5, which was significantly lower than that in menopausal women (median pH 6.0, *Padj* <0.004, pairwise Wilcox-Test) but significantly higher than in pregnant women (median pH = 5.0, *Padj* <0.006, pairwise Wilcox-Test, Table 1; Fig. S1a). No significant difference in cervicovaginal pH was observed among cervical lesions (*P* = 0.772, Kruskal–Wallis test, Table 2).

### Microbial community composition

**Fig 1:**
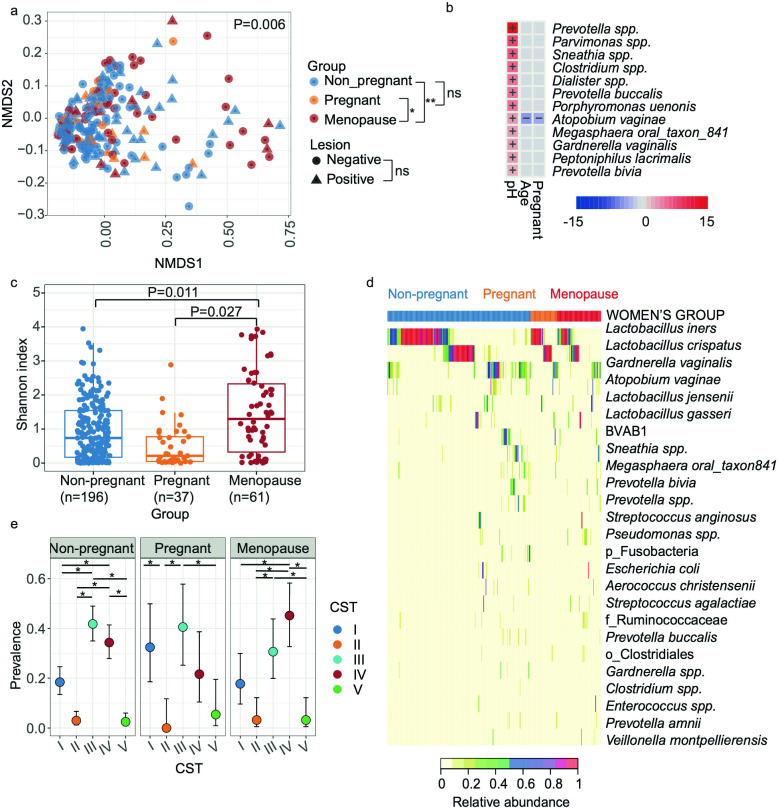
The cervicovaginal microbiota at the species level in nonpregnant, pregnant, and menopausal women were compared. (**a**) Beta diversity with unweighted UniFrac distance. Analysis was performed with PERMANOVA. (b) The heatmap summarizes all the significant relationships between microbial taxa and sample metadata. Color key: -log (*q*-value) * sign color key (coefficient). Cells indicating significant associations are colored (red or blue) and overlaid with a plus (+) or minus (−) sign indicating the direction of the association. (c) Shannon diversity among women groups, analysis was performed with Linear model. (d) Heatmap showing the 25 most abundant taxa, ordered by women’s group and hierarchical clustering within women’s groups. (e) Prevalence of CSTs by women’s group. Analysis was performed with Fisher’s exact test (*P < 0.05, **P < 0.005). Panels created in *R* (see Materials and Methods); and final multi-panel figure mounted using Illustrator Adobe Inc. (2021) (https://adobe.com/products/illustrator).

**Fig 2:**
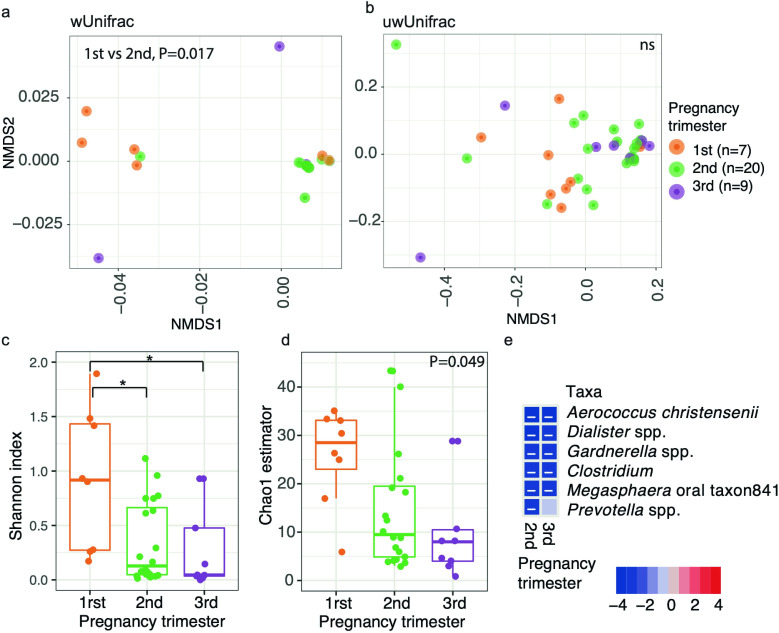
Cervicovaginal microbiota at the species level changes with pregnancy trimesters. (**a and b**) Beta diversity for (a) weighted and (b) unweighted UniFrac distances. Analysis was performed with PERMANOVA. (c** and d**) Alpha diversity for Shannon index (c**C**) and Chao1 estimator (d). Analysis was performed with Linear model. (e) Heatmap of taxa significantly associated with pregnancy trimester analyzed with Maaslin2. The heatmap summarizes all the significant relationships between microbial taxa and sample metadata. Color key: -log (*q*-value) * sign color key (coefficient). Cells indicating significant associations are colored (red or blue) and overlaid with a plus (+) or minus (−) sign indicating the direction of the association, being the first trimester the reference. *P < 0.05, ns = not significant. Panels created in *R* (see Materials and Methods); and final multi-panel figure mounted using Illustrator Adobe Inc. (2021) (https://adobe.com/products/illustrator).

The differences in composition and structure of cervicovaginal microbial communities were evaluated using PERMANOVA models. To avoid collinearity between cervicovaginal pH and women’s group, cervicovaginal pH was only included in models when stratified by women groups. We found that cervicovaginal microbiota composition significantly differed among women’s group (significant for unweighted, *P* = 0.003; generalized, *P* = 0.007; and weighted UniFrac, *P* < 0.030; *R* ^2^ = 0.029–0.032). We observed significant differences in the microbiota between menopausal and pregnant subjects (*Padj* <0.014) as well as between menopausal and nonpregnant subjects [*Padj* <0.038, for unweighted, and weighted UniFrac (Fig. 1a), and generalized UniFrac (not shown)]. The presence of cervical lesions or HPV infections did not significantly influence microbial composition among all women *(P* > 0.050, *R* ^2^ <0.01). For the stratified analysis among nonpregnant or menopausal subjects, cervicovaginal pH was significantly different (*P* < 0.01 among nonpregnant, for all metrics; *P* = 0.046 only for unweighted UniFrac). Finally, for pregnant women, the microbial composition also differed by pregnancy trimester (for all distances, *P* < 0.034, *R* ^2^ >0.13), where first and second trimesters significantly differed (*P* < 0.007, *R* ^2^ = 0.15, pairwise PERMANOVA, for all distances, Fig. 2a and b). We also did a higher rarefaction of 5,000 reads per sample and found similar results (Fig. S2a through e). To provide a comprehensive analysis, we included data analyzed using both SILVA 138 and the GreenGenes extended database. A comparison of the results is presented in Fig. S67, with similar outcomes observed with both databases. However, we found no significant reduction of alpha diversity with the pregnancy trimesters when using SILVA compared to the significant result using GreenGenes. Apart from this, all diversity metrics were consistent between the two databases. In addition, we observed that GreenGenes extended database proved to be better for resolving Lactobacillus species in the cervicovaginal environment.

### Association of bacterial taxa with women’s group, cervicovaginal pH, HPV infections, and cervical lesions

We also analyzed the taxa association, including in the model the covariables of interest using Maaslin2 algorithm, which relies on linear models. We found that overall, *Prevotella, Parvimonas* spp*., Sneathia, Clostridium, Dialister, Prevotella buccalis, Porphyromonas uenonis, Megasphaera oral taxon841, Peptoniphilus lacrimalis, Gardnerella vaginalis, Atopobium vaginae,* and *Prevotella bivia* had significantly greater proportions in less acidic cervicovaginal pH (*Padj* <0.050, Fig. 1b; Table S3). Also, being pregnant or younger was associated with lower *Atopobium vaginae* (Fig. 1b; Table S3). A separate analysis was performed for pregnant women by pregnancy trimester and observed that *Aerococcus christensenii, Dialister spp., Gardnerella spp., Clostridium, Megasphaera oral taxon841* decreased in proportion consistently in second and third when compared to the first trimester, and *Prevotella* spp. decreased in the second trimester (*Padj* >0.046, Fig. 2e; Fig S2i). In general, anaerobic opportunistic taxa were positively associated with higher pH, and negatively associated with age and pregnancy.

We did not detect any bacterial taxa significantly associated with HPV infection or cervical lesions (*Padj* >0.104, Maaslin2). However, before *P*-value adjustment, we observed that two taxa, *Corynebacterium* spp. and *Methanobrevibacter* spp., showed increased proportions in low-risk HPV infections compared to negative samples (*P* = 0.006, and *P* = 0.030, respectively). Also, the two taxa were associated with HR-HPV infections before the *P*-value adjustment for multiple comparisons: *Ureaplasma* decreased while *Clostridium* spp. increased in proportion (*P* = 0.035, and *P* = 0.043, respectively), and, for cervical lesions *Escherichia coli,* increased with LGSIL (*P =* 0.007; Maaslin2). Although marginally, HPV infections and cervical lesions were associated with an increase of the relative abundance of anaerobic-opportunistic taxa, and the significance of these associations may increase with a larger cohort size.

### Alpha diversity varies by group and HGSIL

We used linear mixed models (LMM), including the same covariables of interest as we employed for the beta diversity and taxa association analyses. We found that women’s group (*P* < 6.2e-5) and cervicovaginal pH (*P* < 0.002) were significantly associated with alpha diversity (Fig. S1c). Within women’s group, menopausal subjects showed greater Shannon diversity than nonpregnant [*Padj* <0.010, least-squares means (EMM) for Shannon and Chao1] and then pregnant women (*Padj* = 0.027, only for Shannon, EMM) with no differences between nonpregnant and pregnant subjects (Fig. 1c). However, no significant alpha diversity differences were observed between cervical lesion severity levels.

In the stratified analyses for each women’s group, we found that for nonpregnant group, alpha diversity was greater at higher cervicovaginal pH or age, as well as in the presence of cervical lesions (HGSIL or LGSIL) compared with healthy subjects [*P* < 0.050, pairwise *Padj* <0.050, for Shannon (Fig. S1b), and Chao1, *P* = 0.004, Padj <0.003, EMM]. The later comparisons within pregnant (Padj >0.390, Fig. S1c) or menopause women groups were not different (*P* > 0.450, Fig. S1d). Instead, HPV infection did not significantly influence alpha diversity among all women or nonpregnant women (*P* > 0.050, LMM).

A similar analysis was performed per trimester of pregnancy, initially showing no significant association (*P* > 0.050, for Shannon and Chao1). Nonetheless, when the two outliers were top-coded (that means when data points > 2 x standard deviation away from the mean within each trimester were assigned the last highest value), the pregnancy trimester variable showed a significant trend of decreasing late in pregnancy (*P* < 0.049, for both alpha metrics). Pairwise analysis showed that first trimester was significantly greater than the second and third trimester (*P* < 0.010, for Shannon diversity, not significant for Chao1 estimator, Fig. 2c and d). Indeed, when considering a 5,000 read rarefaction level we found that only the Chao1 estimator evidenced a significant decrease in richness from the first to the third pregnancy trimesters (*P* = 0.01, Fig S2f and g).

In general, alpha diversity analyses showed the greatest values in women with cervical lesions or menopause, and was lowest in pregnant women, particularly in the last trimester of pregnancy.

### Women living in PR are dominated by diverse microbial Community State genotypes

We also evaluated the Community State genotypes (CST) distribution among women’s groups. CSTs are categories that allow classifying each woman’s cervicovaginal microbial composition. In general, they are defined by the dominance of *L. crispatus* (CST-I), *L. gasseri* (CST-II), *L. iners* (CST-III), *L. jensenii* (CST-V), or non*Lactobacillus* dominated (CST-IV) (12). A finer classification by CST subgroups has also been proposed and used in this analysis (18). We used Fisher’s exact test to test the prevalence of these CSTs among women’s groups. As a side note, when analyzing the sequences, only a small percentage (0.61%) of the *Lactobacillus* ASVs were unable to be identified at the species level, indicating that our data for this analysis are reliable. We observed that most women had *Lactobacillus*-dominated CSTs (64.9%), prevailing CST-III (39.5%). No significant differences were observed among women’s groups (*P* = 0.125). However, when stratifying by women’s group, nonpregnant women had a higher prevalence of CST-III (42.3%) followed by CST-IV (34.7%) than any other CST (*Padj* <0.010, Fig. 1e). Instead, pregnant women showed higher CST-III (40.5%) and CST-I (32.4%, *Padj* <0.010, Fig. 1e), with a higher prevalence of the protective *L. crispatus* (Fig. 1d). Nonpregnant and menopause women had mostly *L. iners* and CSTIII and CST-IV (Fig. 1d). Indeed, for menopausal women, CST-IV (45.9%) followed by CST-III (29.5%) were the most prevalent (*Padj* <0.010, Fig. 1e). Among the CST-IV, the subtype CST IV-C was strongly higher in menopausal women (24.6%) than in the other women’s groups (<4.1%, *Padj* = 0.002, Table 4). This profile is characterized by a low relative abundance of *Lactobacillus spp., G. vaginalis, A. vaginae*, and BVAB1. Instead, a diverse array of facultative and strictly anaerobic bacteria exists. Among the different CST IV-C subtypes, menopausal women showed a higher prevalence of CST IV-C0 (11.5%) which is an even microbial profile with a moderate amount of *Prevotella*, and CST IV-C1 (9.8%) which is a *Streptococcus*-dominated profile (Table 4; Fig. S1d).

**TABLE 4:** Distribution of CST categories among women’s group

Women’s group, *n* (%)					
	Nonpregnant	Pregnant	Menopause	TOTAL	
CST categories	(*N* = 196)	(*N* = 37)	(*N* = 61)	(*N* = 294)	*P value^a^*
I	34 (17.3)	12 (32.4)	11 (18.0)	57 (19.4)	*0.161*
I-A	27 (13.8)	10 (27.0)	6 (9.8)	43 (14.6)	*0.071*
I-B	7 (3.6)	2 (5.4)	5 (8.2)	14 (4.8)	*0.449*
II	6 (3.1)	0 (0)	2 (3.3)	8 (2.7)	*0.746*
III	83 (42.3)	15 (40.5)	18 (29.5)	116 (39.5)	*0.301*
III-A	59 (30.1)	15 (40.5)	10 (16.4)	84 (28.6)	*0.021 Padj = 0.084*
III-B	24 (12.2)	0 (0)	8 (13.1)	32 (10.9)	*0.034 Padj = 0.109*
IV	68 (34.7)	8 (21.6)	28 (45.9)	104 (35.4)	*0.057*
IV-A	1 (0.5)	0 (0)	0 (0)	1 (0.3)	*1.000*
IV-B	59 (30.1)	7 (18.9)	13 (21.3)	79 (26.9)	*0.226*
IV-C	8 (4.1)	1 (2.7)	15 (24.6)	24 (8.2)	*1e-7 Padj = 2e-4*
IV-C0	2 (1.0)	1 (2.7)	7 (11.5)	10 (3.4)	*0.001 Padj = 0.009*
IV-C1	4 (2.0)	0 (0)	6 (9.8)	10 (3.4)	*0.014 Padj = 0.075*
IV-C2	2 (1.0)	0 (0)	1 (1.6)	3 (1.0)	*0.700*
IV-C3	0 (0)	0 (0)	1 (1.6)	1 (0.3)	*0.330*
V	5 (2.6)	2 (5.4)	2 (3.3)	9 (3.1)	*0.448*

^*a*^ Fisher’s exact test. *P* value adjustment with "False Discovery Rate" method. Analyses were performed by row, setting in the contingency table positive and negative cases for a particular CST. The negative cases consisted of all subjects who were not assigned to the evaluated category.

**Fig 3:**
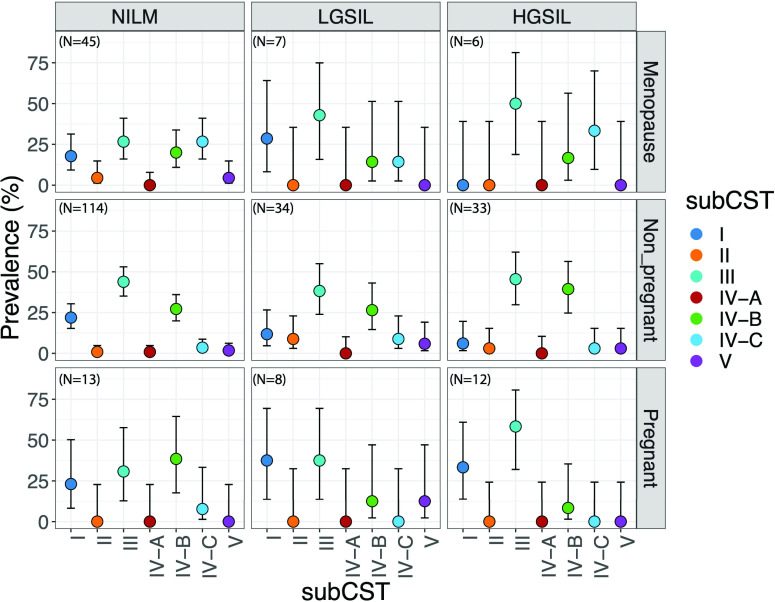
Community State type (CST) distribution by cervical lesion genotypes among women groups. Bars correspond to 95% confidence intervals. CST prevalence was obtained by calculating the 100% among each women group and lesion type. Sample sizes are written in the left-hand upper corner of each box.

In terms of cervical lesions (presence and types), analyses performed with all women by CST had no significant association (*P* < 0.393, Fisher’s exact test, Table 2), nor when stratifying by pregnant, nonpregnant, or menopausal women (Fig. 3; Fig. S4). However, the prevalence of CSTs was found to be significantly different among cervical lesions within nonpregnant women (positive/negative*, P* = 0.033, Table S4), where the presence of cervical lesion showed lower CST-I prevalence compared to its absence, although not significantly (8.7% vs 22.3%, Fisher’s exact test, *Padj* >0.137, data not shown). Analyses of subCSTs within nonpregnant women suggest that women negative for lesion showed higher prevalence of *Lactobacillus*-dominant groups, specially CST I-A (*L. crispatus*-dominated) and CST-III-A (*L. iners*-dominated), although not significantly (Fig. S4). Instead, women with HGSIL showed higher prevalence of CST IV-B (characterized by having a high to the moderate relative abundance of *G. vaginalis* with some *Atopobium vaginae*). However, this difference was also not statistically significant. CST-IV is characterized by markers of cervicovaginal dysbiosis (Fig. S4).

We found no significant differences (*P* < 0.05, Fisher’s exact test**,** data not shown) in CSTs with HPV infections (presence and types) among all women. However, when considering only nonpregnant women, we did find significant differences between HR-HPV/Only_LR-HPV/negative (*P* = 0.039, Fisher’s exact test, Table S5), which shows that women with LGSIL had a particularly low prevalence of CST-IV compared to HGSIL and negative samples (11.8% vs 35.5% and 43%, respectively, Table S5). In general, being menopausal, having cervical lesions, and being infected with exclusively HR-HPV infections are traits associated with diverse microbial profiles. We also aimed to compare CSTs from nonpregnant Puerto Rican women with those of U.S. Hispanic women (self-declared) reported by Ravel et al. (12). We found no significant differences between Hispanics from the U.S. vs Puerto Rican Hispanics (*P* > 0.132, Fisher’s exact test, Table S6). For example, for CST IV, 38.1% and 34.7%, respectively (*P* = 0.881).

### Most abundant *L. iners*-ASVs were not associated with cervical lesions or HPV infection

We also tested if the top15 most abundant ASVs classified as *L. iners* were associated with cervical lesions or their types. We did not find significance for any *L. iners*-ASVs by cervical lesion or HPV infection, not even by the severity of lesion or HPV risk genotype (*P* > 0.050, Fisher’s exact test, data not shown).

### Longitudinal changes of the cervicovaginal microbiota in a subset of women

To explore whether microbial profiles in this population vary over time, we analyzed two-time points longitudinal data. Thirty-nine women (nonpregnant, *n* = 29; pregnant, *n* = 1; menopausal, *n* = 9) who had had a second sampling between 4 to 16 mo after their first visit were analyzed. Approximately half of women (51.3%, 20/39) kept the same CST between visits, and only 12.8% (5/39) changed from a *Lactobacillus*-dominated (all CST-III) to a diverse profile (IV). The same proportion, 12.8% (5/39), changed from a diverse (CST-IV) to *Lactobacillus*-dominated profile (CST-I and II). A total of 35.9% (14/39) of women changed between different combinations of *Lactobacillus*-dominated CSTs (CST-I, II, and III), with no particular trends and with no differences among women’s groups (Fig. S5a and b). Cervical lesion phenotype was maintained between the two visits for 56.4% (22/39) of women. Meanwhile, HPV status coincided in 67.6% (25/37) of women from the first to the second visit (Fig. S5c and d). Changes in CST based on cervical lesions or HPV infection were also explored; however, sample sizes were too small to perform a reliable comparison. Transitions from *Lactobacillus*-dominated to non*Lactobacillus*-dominated profiles or vise-versa did not show any significant trend for cervical lesions or HPV status (*P* < 0.225, Fisher’s exact test, Table S7 and S8).

## DISCUSSION

This study revealed not only an abundance and ubiquity of CST III and IV of the cervicovaginal bacterial microbiota but also a different distribution of oncogenic HPVs in this Puerto Rican cohort. The most frequent genotypes were HPV51, 16, 33, 52, and 56, mostly in HGSIL lesions, with few to no HPV18. This seems to indicate that both the quadrivalent HPV vaccine (4vHPV) (targeting HPV genotypes 6, 11, 16, and 18) and the 9-valent HPV vaccine may have limited effectivity as they lack coverage of HPV51 and 56, HPV genotypes that are prevalent in our cohort. The role of multiple HR-HPV infections, specifically HPV51 and 56, in the aggravation of cervical lesions has not been studied in PR and deserves further attention. We found that women in menopause had the lowest vaccination prevalence (4.9%), and this is expected as vaccination in younger women has better promotion and adherence. In PR, HPV vaccination started at time of HPV vaccine approval in USA back in 2006. The vaccination focusses at the time on the younger population, and many were vaccinated after being sexually active. Thus, the low rate of vaccination in this cohort.

The prevalence of HPV in this study was similarly high (74.8%; HR-HPV, 67.3%) when compared to that reported in a previous study in the Puerto Rican population by our team (84%; HR-HPV, 79%) (16), as well as in Hispanic and Amerindian populations (77%; HR-HPV, 65.9%) (19). The prevalence of HPV in PR was higher than that observed in other Hispanic populations, including Costa Ricans (carcinogenic, 35%) (20), Europeans (~20%) (21), Japanese (~20%) (22), and Nigerian (carcinogenic, 40%) (23), using the same SPF10/LiPA25 HPV detection method. The high prevalence of HR-HPV genotypes in our cohort is however not generalizable to the population of PR, as clinics cover general gynecology and colposcopy, that is, some patients are referred for colposcopy, hence the higher prevalence of HR-HPVs. In the past, a population-based study in PR showed that the prevalence of HR-HPV genotypes was lower; however, they included only HPV types 6, 11, 16, and 18 and used a different detection method (24).

Although PR is a Caribbean country, it differs from other Afro-Caribbean women from Barbados, where the cervicovaginal microbial community is mostly dominated by non*Lactobacillus* genera (72%), including *Prevotella* (25). This is possible due to differences in African ancestry between Barbados and PR, as well as disparities in their lifestyle. The cervicovaginal microbiota of women living in PR was dominated by *L. iners* (39.5%) or diverse non*Lactobacillus* communities (35.4%). This is consistent with previous work from our lab for Puerto Rican population (16), U.S. Hispanics (12), and Venezuelans (26).

Both *L. iners*-dominant profiles and high diversity profiles (i.e., CSTs III and IV in ~77% of nonpregnant women and in menopausal women) have been associated, in previous studies, with bacterial vaginosis (27), STI infections (28, 29), and adverse pregnancy outcomes (30, 31). Although *L. iners* is a *Lactobacillus* species, its metabolic and ecological characteristics make it the least protective cervicovaginal *Lactobacillus*. Unlike other health-associated *Lactobacillus*, *L. iners* can cohabit in a diverse environment and act as a vaginal symbiont, or as an opportunistic pathogen if surrounded by other anaerobic pathogens (32). Its H_2_O_2_ production is null or very limited (33, 34). It also does not promote a sufficiently acidic environment that inhibits undesirable bacteria (35). Additionally, it generates only L-lactic acid instead of the protective D-lactic acid isomer. This promotes inflammation (36) and facilitates invasion of the cervix by opportunistic bacteria (37). Diverse cervical microbial profiles have also been associated with high levels of pro-inflammatory genital cytokines. This damages the endocervix’s columnar epithelial barrier, exposing it to the colonization of other microbial or pathogenic agents (38, 39). Hence, the existence of these two microbial profiles in the Puerto Rican population may promote higher susceptibility toward disorders in the cervicovaginal area and inflammation conditions (40).

Diverse microbial profiles (CST-IV) in our nonpregnant population were mostly represented by CST-IV-B (30.1%), like Hispanic women in the United States (18). This profile is considered of higher risk for bacterial vaginosis since it includes taxa such as *Gardnerella vaginalis* (41) that are associated with an increased risk of viral infections including HPV (28, 42 - 45). Menopausal women, besides having this profile (21.3%), also had a high prevalence of CST-IV-C (24.6%), with some women being high in *Prevotella* (CST-IV-C0) and others in *Streptococcus* (CST-IV-C1), as previously seen in other menopause studies in U.S. population (18, 46). Although CST-IV-C is a non*Lactobacillus*-dominated profile, it has been associated with the lowest Nugent scores (Gram stain scoring system to diagnose bacterial vaginosis, where lower values suggest no B.V.). However, CST-IV-C high in *Prevotella* has been shown to induce a significantly greater pro-inflammatory signaling (38) and is considered unfavorable for menopausal women, particularly those with cervical lesions or infected by HPV. During menopause, significant hormonal changes occur. The decline in estrogen level can lead to a decrease in the cervical mucus (vaginal dryness) and glycogen deposition, the primary source of *Lactobacillus* (47). Low *Lactobacillus* induces high cervicovaginal pH leading to an environment more susceptible to infections (48).

Contrary to menopause, pregnant women showed a decrease in microbial alpha diversity (particularly later in pregnancy) and an increase in *Lactobacillus*-dominated profiles (CST-III and CST-I), a phenomenon already reported by other groups (49). This conversion of high to low alpha diversity is accentuated in pregnant Hispanic and Black populations (50), two racial groups with high cervicovaginal microbial diversity. Variation in cervicovaginal community composition during pregnancy may also correspond with hormonal changes. The increase in estrogen levels promotes higher glycogen deposition in cervicovaginal epithelial cells (51). *Lactobacillus* spp. utilize glycogen as the primary carbohydrate source to produce lactic acid, contributing to the protective effect of a low cervicovaginal pH (lysing bacteria other than *Lactobacillus*). Additionally, the dominance of *Lactobacillus* produces significant amounts of bacteriocins, contributing to colonization resistance (11, 52 - 54). This defensive strategy explains the reduction of *Gardnerella* spp., *Atopobium vaginae,* and *Prevotella* spp. observed in our study, which has also been reported in other pregnant Hispanics (50). Moreover, our study identified a reduction in other BV-associated taxa, such as *Aerococcus christensenii, Megasphaera spp., Dialister spp.,* and *Clostridium*. The lower abundance of *Atopobium vaginae* observed in pregnant women, as compared to women in menopause or the nonpregnant group, can be associated with the lower pH found during pregnancy which reduces the colonization of these BV-associated species. To note, in our pregnant cohort, no women had the CST-III-B type, only CST-III-A (40.5%). CST-III-A is associated with a lower Nugent score and cervicovaginal pH (18), indicating greater protection against infection.

Previous studies have shown that greater microbial diversity in cervicovaginal samples and a community profile dominated by *L. iners-* are both associated with an increased risk of HPV infection and persistence (46, 55 - 57). However, in our study, very little or no association was detected. Differences with other studies may be explained by ethnicity or the HPV detection method. However, this lack of a clear HPV-microbial association has been reported in other Hispanic populations with similar profiles and using the same HPV detection method (16, 26). This might be due to the high prevalence of HPV and a higher dominance of diverse and *L. iners*-dominated profiles in our cohort, as compared to Caucasian women with *L. crispatus-*dominated profiles. Additionally, differences between transient and persistent HPV infection may influence this association since only persistent infections have been associated with immune response and cervicovaginal dysbiosis (58). Chronic cervical inflammation caused particularly by diverse bacterial populations, in addition to HPV persistence, creates a favorable context for neoplasia and later cancer development (59, 60). However, longitudinal methods to define HPV persistence, especially using HPV typing methods with higher analytical sensitivity, are expensive and time-consuming, and therefore studies are scarce, but very relevant.

We found greater Shannon diversity in HGSIL and LGSIL and a lower prevalence of *L. crispatus*-dominated profiles (higher of CST III, IV-B, and IV-C) when compared to healthy subjects, which supports previous findings (16, 61, 62). Additionally, women with LGSIL tend to have coinfections with both HPV genotypes (HR and LR-HPV), while those with HGSIL showed a greater prevalence of infections with exclusive HR-HPV types. The coexistence of HR and LR-HPV risk genotypes was similarly prevalent in HR-HPV or LR-HPV exclusive infections, a finding that has also been observed in Hispanic-Venezuelan populations (19). This association has previously been linked to sexual partner turnover (63) and a lower risk of cervical cancer development (64 - 66).

We also explored the temporal stability between cervical lesions, HPV status, and cervicovaginal microbiota in a small subset of women. Changes observed between positive and negative lesions or HPV infections may be due to treatment (loop electrosurgical excision procedure, LEEP, or endocervical curettage, ECC) or natural clearance. For the cervicovaginal microbiota, we found no specific trends between CST changes over time nor concerning HPV infection and cervical lesion. However, previous studies have demonstrated that CST-IV-B (with high to moderate *G. vaginalis* and *Atopobium vaginae*) and CST-III are more likely to transition to other CSTs (32, 67) or that CST-IV-B often transitioned to CST-III (68). Limitation of this analysis includes the small sample size and the long period between study visits for these patients. CST transition can be very rapid, for example, switching for only a single day (67). Furthermore, our community has a deficit in *L. crispatus*-dominated profiles, which restricts the examination of transitional trends from, or toward, this health-related microbial pattern.

In conclusion, women living in PR, regardless of their physiological stage, predominantly maintained cervicovaginal communities dominated by *L. iners* (CST III) or a diverse microbial profile (e.g., CSTs IV-B and IV-C). Even though these profiles have been previously associated with reduced cervicovaginal health, they were not found to be significantly linked to cervical lesion in our cohort. However, the high prevalence of HR-HPV and the presence of these less stable, nonoptimal bacterial profiles may be associated with the greater risk of cervical cancer observed in our populations. As a result, Puerto Rican women may be considered a susceptible population for cervical lesion development.

## MATERIALS AND METHODS

### Study design and participant sample collection

This cross-sectional study of adult women coming for gynecology and colposcopy evaluation at the UPR and San Juan City clinics (San Juan Puerto Rico, Metropolitan area), who did not meet the exclusion criteria, were recruited to participate in this study. *Exclusion criteria included:* (1) history of regular urinary incontinence; (2) treatment for or suspicion of prior toxic shock syndrome; (3) candidiasis; (4) active urinary tract infections; (5) active STIs; and (6) cervicovaginal irritation at the time of screening. Only asymptomatic women were included. Clinicians do not test for STIs in the asymptomatic population as these are not routine. We selected these exclusion criteria based on the indications from the Manual of Procedures of the Human Microbiota Project protocol (69). However, we included women who took antibiotics during the last 2 mo, since they were few (7.1%) and were distributed similarly among the women groups.

The date range in which human subjects’ data/samples were collected was between November 2017 and February 2020. The study and its procedures were conducted from March 2020 to September 2022. The study was approved by the Ethics Committees of the UPR-Medical Sciences Campus IRB (Protocol ref. 1050114/June 2014), San Juan City Hospital and has biosafety approval protocol # 94620. All subjects were informed (both verbally and in writing) of the sampling procedure, risks, and benefits of the study, gave written informed consent and signed HIPAA forms, following the Declaration of Helsinki. All staff involved in the project were certified by: CITI RCR, Social and Behavioral Research Best Practices for Clinical Research, HIPAA certifications and the NIH training on Protection of Human Subjects. Patients completed a metadata questionnaire with demographic characteristics (age, place of birth, employment, educational attainment), assessment of sexual risk (including the age of onset, current sexual partners), health history, antibiotic use, vitamins, and BMI.

A total of 367 samples were collected from which 337 were successfully sequenced and after rarefaction 4 samples were removed for low sequence count, for a total of 333 samples which corresponds to 294 women. Samples were collected using sterile Catch-All Specimen Collection Swabs (Epicentre Biotechnologies, Madison, WI), and placed in MoBio bead tubes with buffer (MoBio PowerSoil kit, MoBio, Carlsbad, CA) (69). Swabs were then swirled for ~20 s in 750 µL of MoBio buffer in the labeled specimen collection tube. For the sampling, a speculum was inserted for access and visualization of the cervix. The sterile swab was placed in the posterior fornix (cervix) and rotated along the lumen with a circular motion and swabs were immediately placed in the MoBio tubes. The area of the posterior fornix was used as a sample site to improve the detection of HPV and as a reliable indicator of the overall cervicovaginal environment, which has been demonstrated in previous research (70). Besides swabs, we collected approximately 10 mL of cervical lavages by injecting PCR-grade sterile water into the vaginal canal for future studies. Collected lavage was transferred to a clean 15 mL collection tube and pH was measured with hydrion wide range pH paper strips. All samples were coded and placed in ice up to 4 h. Subsequently, the samples were transported to the laboratory and stored at −80°C until nucleic acid extraction and PCRs were performed at a single laboratory (FGV) to minimize processing variation.

### DNA extraction and 16s rRNA sequencing

Genomic DNA extraction was done on posterior fornix (cervical) swabs using Qiagen Power Soil Kit (QIAGEN LLC, Germantown Road, Maryland, USA). No human DNA sequence depletion or enrichment of microbial or viral DNA was performed. A detailed description of the optimized extraction protocol is available in a previously published study (16). In short, Powerbead tubes were homogenized for 10 min at 3,200 rpm, using the Vortex-Genie 2, G560 (Scientific Industries, Inc. NY). We combined 100 µL of solution C2 and 100 µL of solution C3 and vortexed for 5 s for cell lysis, and the elution solution was 100 µL sterile PCR water, which was warmed to 55°C, and to increase DNA yield, allowed to remain on the filter for 5 min before the final centrifugation step. DNA concentration was measured using the Qubit dsDNA H.S. (High Sensitivity) Assay (Waltham, Massachusetts, U.S.) (ranging from 5 to 100 ng/µL). Genomic DNA from samples was shipped to an outsourced sequencing facility with an average genomic DNA concentration of 10–30 ng/µL. No human DNA sequence depletion or enrichment of microbial DNA was performed.

The DNA obtained from cervical samples was normalized to 4 nM during 16S rRNA gene library preparation. Universal bacterial primers, 515F (5’GTGCCAGCMGCCGCGGTAA3’) and 806R (5’GGACTACHVGGGTWTCTAAT3’) as in the Earth Microbiota Project (http://www.earthmicrobiota.org/emp-standard-protocols/16s/), were used to amplify the hypervariable V4 region of the 16S ribosomal RNA marker gene (~291 bp) (71). The methodology applied for this process was identical to the one implemented in previous reports (16, 72 - 74). Amplicons were quantified using PicoGreen (Invitrogen) and a plate reader (Infinite 200 PRO, Tecan). Once quantified, volumes of each of the products were pooled into a single tube so that each amplicon is represented in equimolar amounts. This pool was then cleaned up using AMPure XP Beads (Beckman Coulter), and quantified using a fluorometer (Qubit, Invitrogen). Customized sequencing was outsourced at Argonne National Laboratory (Illinois, USA) using Illumina MiSeq with the 2 × 250 bp paired-end sequencing kit. Amplicons were sequenced with the MiSeq Illumina platform in three different runs. The facility adds a negative control, and nothing is reported if they don’t produce sequence reads above 500 total reads. Internal positive controls are analyzed and aligned to the sequencer in real-time.

The reads obtained from the 16S-rRNA sequencing and corresponding metadata were uploaded in QIITA (75) study ID 12871 (https://qiita.ucsd.edu/study/description/12871). In addition, the resulting raw sequences were made available at the European Nucleotide Archive Project (ENA) under the access number ERP136546.

### HPV genotyping and cytologic diagnoses

The kit used to complete the HPV genotyping consists of a highly sensitive short-polymerase chain reaction-fragment assay (Labo Biomedical Products, Rijswijk, The Netherlands, licensed Innogenetics technology). This process takes advantage of SPF10 primers to amplify a 65 bp fragment contained at the L1 open reading frame of HPV genotypes, followed by a reverse-hybridization reaction to determine which HPV genotypes are present in the sample by comparing the results to standardized controls provided by the kit. This assay facilitates the identification of the following mucosal HPV genotypes: 6, 11, 16, 18, 31, 33, 34, 35, 39, 40, 42, 43, 44, 45, 51, 52, 53, 54, 56, 58, 59, 66, 68, 73, 70, and 74 and classified as either high-risk (HR-HPV) or low-risk (LR-HPV). This methodology is explained thoroughly in a previously published study (16). HPV genotype groups categorized samples as (1) only HR-HPV (exclusively HR types), (2) only LR-HPV exclusively low-risk HPVs, (3) both (if a sample contained both HR and low-risk HPV genotypes, (4) HPV negatives or (5) missing (no HPV genotyping done on these samples) (Table 1).

Data from the medical records and questionnaires were obtained at the time of patient visit and reviewed for results of cervical cytology and pathology reports. Data regarding abnormal cervical cytology were classified according to Bethesda system (76) in atypical squamous cells of undetermined significance (ASCUS), atypical squamous cells, cannot exclude high-grade lesion (ASCH-H), low-grade squamous intraepithelial lesion (LGSIL), high-grade intraepithelial lesion (HGSIL), or squamous cell carcinoma (SCC). Missing values in the different categories corresponded to the fact that no information for a given category was retrieved for that specific participant.

### Sequence processing

Three Illumina demultiplexed paired-end sequence runs were uploaded and run independently in QIIME2. DADA2 algorithm was used for truncating sequences based on quality, merging sequences, amplicon error correction, chimera identification, and obtaining representative sequences (features). Sequence length truncation was performed separately for each run, just before the drop of the base qualities. The maximum length truncation was 15 bases (reverse sequence). Taxonomy classification was performed using a Naïve Bayes classifier implemented in the q2-feature-classifier plugin (77) against a custom GreenGenes modified database that contains sequences from GreenGenes, the Human Oral Microbiota Database, and cervicovaginal vaginal reference sequences from the Human Microbiome Database (78) used previously (79 - 81). Feature tables for the three runs were finally merged. Samples showed and average of 26,308 sequences/sample [min. 187 sequences, max. 80,253 sequences]. Rarefaction was run at 1,773 sequences per sample to include most of the samples, and four samples with a lower sequence number were eliminated (Table S1). All analyses were run with the rarefied table at the species level. An additional rarefaction of 5,000 sequences per sample was also run and feature tables were analyzed in parallel. For this threshold, a total of 16 samples were lost. Results between the two rarefaction cutoffs were similar. We also re-analyzed our data using the SILVA 138 database following the exact same parameters and metrics (see Fig. S6 and S7), also with similar results. The effect of the “Sequencing run” was also included in all models of the analysis to account for the possible batch variability; however, no significant differences were observed.

### Statistical analysis

Population description was performed comparing the three main groups: nonpregnant, pregnant, and menopausal women. Categorical variables were compared with Fisher’s exact test and continuous variables with the Kruskal–Wallis test.

Cytology smears were performed for cervical lesion diagnosis; however, some women required biopsy. Therefore, a consensus result was used for the analyses. Only one sample was diagnosed as squamous cell carcinoma (SCC) and it was included as HGSIL to facilitate the analysis. Additionally, diagnosis with ASCUS (5.4%, 14/258) was top-coded as LGSIL if they were HPV positive and negative when HPV negative. Then, the variable “Type of cervical lesions” only included LGSIL, HGSIL, and negative samples.

Comparison among HPV genotype categories first explored the distribution of women with the presences of any HR-HPV, as well as those with only LR-HPV genotypes.Subsequently, the distribution of women infected exclusively with HR-HPV or LR-HPV or mixed infections, indicating the presences of HR- and LR-HPV genotypes (called “both” on Tables 1 and 2), was examined. Another comparison was also performed for mixed infections, although this one includes “Number of HR-HPV genotypes” or “Number of LR-HPV genotypes,” referring to the actual number of different HPV genotypes detected in a particular sample.

### Beta diversity

Beta diversity was evaluated using unweighted, generalized, or weighted UniFrac distances. Collinearity among variables was checked with Spearman’s correlation using “cor.test” R function before including variables to the model. These included the following:

distance ~Group (nonpregnant/pregnant/menopause) +Type of cervical lesion (LGSIL/HGSIL/negative) +HPV genotype (only HR-HPV/ only LR-HPV/ both HPV types/ negative) +BMI (numerical variable) +Antibiotics last 2 mo (yes/no) +Sequencing run (Run A/B/C).

The trimester of pregnancy was also included only among pregnant women. For the stratified analysis for each of the women groups, the Age variable was also included. The variable “Sequencing run” was set as a random variable to account for the variability among runs.

Variables included in the model were analyzed using nonparametric permutational multivariate analysis of variance [PERMANOVA (82)] with “adonis2” function from "vegan" R package (83). Permutation was set to 1000 and seed was set to 711. PERMANOVA model allows comparing variance between groups to the variance within groups (spatial location differences). “Run” was set as a random variable using “setBlocks” function from the “permute” R package (84).

Pairwise analyses for the significant categorical variables were performed with “pairwise.adonis2” function from the “pairwiseAdonis” R package (Martinez Arbizu 2017).

### Taxa association with study variables

Microbial taxa and their association with all the above variables were assessed using Maaslin2’s linear model in R (85), where we also set “Sequencing run” as random variable. Maaslin2 is a bioinformatic tool that helps to identify taxa-variables associations. The output table (Table S3) shows the specific taxa associated with a particular variable. The table also provides the model coefficient value (effect size), the *P*-value, and the *P*-value adjusted for false discovery rate (“fdr”), among other parameters. If the variable is categorical, a reference is needed; negative samples were always set as the reference for our analysis. A positive coefficient indicates a positive association (increase in a taxon abundance) with a particular variable. A heatmap is also generated where the plus sign in the red squares suggests an increase in the taxa abundance for a specific variable, while a negative sign in the blue squares indicates a decrease of abundance, always based on the reference (Fig. 1E). The model included women’s group, HPV types, type of cervical lesions, and significant variables in the beta diversity analysis (pH, age).

### Alpha diversity

Microbial alpha diversity was measured with the Shannon index calculated using “diversity” function from "vegan" R package (83) and the Chao1 estimator calculated using the “apply” function from the “OTUtable” library (86). Shannon index provides information about richness and evenness of a microbial community by sample, while Chao1 estimator provides only the microbial richness estimated in an exhaustive sampling scenario. Linear mixed-effect model (LMM), “lmer” function from “lmerTest” R package (87) were fitted, setting “Sequencing run” as the random variable to account for the differences in sequencing facilities. Fixed (explanatory) variables included were the same as those used in the beta diversity analysis. To obtain the best-fitted model we utilized the “step” function from “lmerTest” R package (87).

The trimester of pregnancy was also included only among pregnant women. For the stratified analysis for each of the women groups, “age” variable was also included. Collinearity among variables was checked with Spearman’s correlation using “cor.test” R function, before including variables to the model. We also checked the normality of the linear regression residuals and did logarithmic transformation to alpha diversity metrics to reach residual normal distribution when necessary. Models were run for between 164 and 184 samples due to missing samples. R library “emmeans” (88) was used to run pairwise analyses for mixed model.

### Community State types

Each women’s sample was classified into CST following the protocol of Valencia program (18) in Python 3 (https://github.com/ravel-lab/VALENCIA). Input data had to be formatted using local scripts. To compare the proportion of CST in women in PR from this study, with a similar Hispanic population but living in the USA, metadata from a very known study of cervicovaginal microbiota were downloaded and compared (12). Prevalence of CSTs was also compared among women groups and among cervical lesions. A 95% confidence interval for these values was calculated using “BinomCI” function from “DescTools” R package (89).

### Longitudinal changes of the cervicovaginal microbiota in a subset of women

To explore whether microbial profiles in this population vary over time, we analyzed two-time points longitudinal data. Thirty-nine women who had had a second sampling between 4 to 16 mo after their first visit were analyzed. We first used the CST classification to group women with CSTs dominated by *Lactobacillus* (CST-I, II, III, and V) or diverse (CST-IV). We then built a table to observe the coincidences, first among type of cervical lesions and then among the positive and the negative HPV infection. Finally, we ran a Fisher’s exact test for each table (Table S7 and S8).

As supplementary materials, we provide the updated STORMS checklist (The Organization and Reporting of Microbiome Studies), which we updated based on our study’s criteria to provide complete reporting of our study and ensure reproducibility.

## Data Availability

Data are available. The reads obtained from the 16S-rRNA sequencing and its corresponding metadata were uploaded in QIITA [76] study ID 12871 ( https://qiita.ucsd.edu/study/description/12871 ). In addition, the resulting raw sequences were made available at the European Nucleotide Archive Project (ENA) under the access number ERP136546 ( https://www.ebi.ac.uk/ena/browser/view/PRJEB51893?show=reads). Supplement data are also available.

## AFTER EPUB

[This article was published on 3 August 2023 with a typographical error in the Discussion. The error was corrected in the current version, posted on 7 August 2023.]

## SUPPLEMENTAL MATERIAL

Fig. S1 msystems.00357-23-s0001.eps Vaginal pH association with women's group.

Fig. S2 msystems.00357-23-s0002.tif The cervicovaginal microbiota in nonpregnant, pregnant, and menopausal women.

Fig. S3 msystems.00357-23-s0003.eps Vaginal microbial association with women's group, age, and vaginal pH.

Fig. S4 msystems.00357-23-s0004.tif Vaginal sub-Community State type distribution among women's group for low-grade squamous intraepithelial lesion (LGSIL), high-grade squamous intraepithelial lesions (HGSIL), and women negative for cervical lesions.

Fig. S5 msystems.00357-23-s0005.eps Changes between two consecutive visits for (a, b) vaginal Community State types (CST) showing the percentage of a particular CST that is maintained or that changes, (c) cervical lesion, and (d) HPV status among all women.

Fig. S6 msystems.00357-23-s0006.tif The cervicovaginal microbiota analysis for GreenGenes 138 extended database and SILVA 138 in nonpregnant, pregnant, and menopausal women.

Fig. S7 msystems.00357-23-s0007.tif Cervicovaginal microbiota analysis for GreenGenes 13.8 extended database and SILVA 138 between pregnancy trimesters.

STORMS checklist msystems.00357-23-s0008.docx An editable version for adaptation and inclusion in publications is available from https://stormsmicrobiome.org.

Supplemental tables msystems.00357-23-s0009.pdf Tables S1 to S8.

ASM does not own the copyrights to Supplemental Material that may be linked to, or accessed through, an article. The authors have granted ASM a non-exclusive, world-wide license to publish the Supplemental Material files. Please contact the corresponding author directly for reuse.

## References

[1] Arbyn M , Weiderpass E , Bruni L , de Sanjosé S , Saraiya M , Ferlay J , Bray F . 2020. Estimates of incidence and mortality of cervical cancer in 2018: a worldwide analysis. Lancet Glob Health 8:e191–e203. doi:10.1016/S2214-109X(19)30482-6 31812369 PMC7025157

[2] Inc., A.C.S . 2021. Cancer facts & figures for Hispanic/Latino people 2021-2023. Available from: https://www.cancer.org/content/dam/cancer-org/research/cancer-facts-and-statistics/cancer-facts-and-figures-for-hispanics-and-latinos/hispanic-latino-2021-2023-cancer-facts-and-figures.pdf

[3] Organization PAH . 2013. World Health Organization regional office for the Americas: Puerto Rico CANCER profile 2013. Available from: https://www.paho.org/en/documents

[4] Ortiz AP , Ortiz-Ortiz KJ , Colón-López V , Tortolero-Luna G , Torres-Cintrón CR , Wu C-F , Deshmukh AA . 2021. Incidence of cervical cancer in Puerto Rico, 2001-2017. JAMA Oncol 7:456–458. doi:10.1001/jamaoncol.2020.7488 33443548 PMC7809611

[5] Ilhan ZE , Łaniewski P , Thomas N , Roe DJ , Chase DM , Herbst-Kralovetz MM . 2019. Deciphering the complex interplay between microbiota, HPV, inflammation and cancer through cervicovaginal metabolic profiling. EBioMedicine 44:675–690. doi:10.1016/j.ebiom.2019.04.028 31027917 PMC6604110

[6] Lehoux M , D’Abramo CM , Archambault J . 2009. Molecular mechanisms of human papillomavirus-induced carcinogenesis. Public Health Genomics 12:268–280. doi:10.1159/000214918 19684440 PMC4654617

[7] Pradhan SR . 2010. Human papillomavirus infections in pregnant women and its impact on pregnancy outcomes: possible mechanism of self-clearance. IntechOpen, 2020.

[8] Wang X , Huang X , Zhang Y . 2018. Involvement of human papillomaviruses in cervical cancer. Front Microbiol 9:2896. doi:10.3389/fmicb.2018.02896 30546351 PMC6279876

[9] Waxman AG , Chelmow D , Darragh TM , Lawson H , Moscicki A-B . 2012. Revised terminology for cervical histopathology and its implications for management of high-grade squamous intraepithelial lesions of the Cervix. Obstet Gynecol 120:1465–1471. doi:10.1097/aog.0b013e31827001d5 23168774 PMC4054813

[10] Pfeiffer JK . 2016. Host response: microbiota prime antiviral response. Nat Microbiol 1:15029. doi:10.1038/nmicrobiol.2015.29 27571982

[11] Zhou Y , Wang L , Pei F , Ji M , Zhang F , Sun Y , Zhao Q , Hong Y , Wang X , Tian J , Wang Y . 2019. Patients with LR-HPV infection have a distinct vaginal microbiota in comparison with healthy controls. Front Cell Infect Microbiol 9:294. doi:10.3389/fcimb.2019.00294 31555603 PMC6722871

[12] Ravel J , Gajer P , Abdo Z , Schneider GM , Koenig SSK , McCulle SL , Karlebach S , Gorle R , Russell J , Tacket CO , Brotman RM , Davis CC , Ault K , Peralta L , Forney LJ . 2011. Vaginal microbiome of reproductive-age women. Proc Natl Acad Sci U S A 108 Suppl 1:4680–4687. doi:10.1073/pnas.1002611107 20534435 PMC3063603

[13] Bryc K , Durand EY , Macpherson JM , Reich D , Mountain JL . 2015. The genetic ancestry of African Americans, Latinos, and European Americans across the United States. Am J Hum Genet 96:37–53. doi:10.1016/j.ajhg.2014.11.010 25529636 PMC4289685

[14] Via M , Gignoux CR , Roth LA , Fejerman L , Galanter J , Choudhry S , Toro-Labrador G , Viera-Vera J , Oleksyk TK , Beckman K , Ziv E , Risch N , Burchard EG , Martínez-Cruzado JC . 2011. History shaped the geographic distribution of genomic admixture on the island of Puerto Rico. PLoS One 6:e16513. doi:10.1371/journal.pone.0016513 21304981 PMC3031579

[15] Silva-Zolezzi I , Hidalgo-Miranda A , Estrada-Gil J , Fernandez-Lopez JC , Uribe-Figueroa L , Contreras A , Balam-Ortiz E , del Bosque-Plata L , Velazquez-Fernandez D , Lara C , Goya R , Hernandez-Lemus E , Davila C , Barrientos E , March S , Jimenez-Sanchez G . 2009. Analysis of genomic diversity in Mexican Mestizo populations to develop genomic medicine in Mexico. Proc Natl Acad Sci U S A 106:8611–8616. doi:10.1073/pnas.0903045106 19433783 PMC2680428

[16] Godoy-Vitorino F , Romaguera J , Zhao C , Vargas-Robles D , Ortiz-Morales G , Vázquez-Sánchez F , Sanchez-Vázquez M , de la Garza-Casillas M , Martinez-Ferrer M , White JR , Bittinger K , Dominguez-Bello MG , Blaser MJ . 2018. Cervicovaginal fungi and bacteria associated with cervical intraepithelial neoplasia and high-risk human papillomavirus infections in a hispanic population. Front Microbiol 9:2533. doi:10.3389/fmicb.2018.02533 30405584 PMC6208322

[17] Auriemma RS , Scairati R , Del Vecchio G , Liccardi A , Verde N , Pirchio R , Pivonello R , Ercolini D , Colao A . 2021. The vaginal microbiome: a long urogenital colonization throughout woman life. Front Cell Infect Microbiol 11:686167. doi:10.3389/fcimb.2021.686167 34295836 PMC8290858

[18] France MT , Ma B , Gajer P , Brown S , Humphrys MS , Holm JB , Waetjen LE , Brotman RM , Ravel J . 2020. VALENCIA: a nearest centroid classification method for vaginal microbial communities based on composition. Microbiome 8:166. doi:10.1186/s40168-020-00934-6 33228810 PMC7684964

[19] Vargas-Robles D , Magris M , Morales N , de Koning MNC , Rodríguez I , Nieves T , Godoy-Vitorino F , Sánchez GI , Alcaraz LD , Forney LJ , Pérez M-E , García-Briceño L , van Doorn L-J , Domínguez-Bello MG . 2018. High rate of infection by only oncogenic human papillomavirus in Amerindians. mSphere 3:e00176-18. doi:10.1128/mSphere.00176-18 29720524 PMC5932372

[20] Safaeian M , Herrero R , Hildesheim A , Quint W , Freer E , Van Doorn L-J , Porras C , Silva S , González P , Bratti MC , Rodriguez AC , Castle P , Costa Rican Vaccine Trial Group . 2007. Comparison of the SPF10-Lipa system to the hybrid capture 2 assay for detection of carcinogenic human papillomavirus genotypes among 5,683 young women in Guanacaste, Costa Rica. J Clin Microbiol 45:1447–1454. doi:10.1128/JCM.02580-06 17344361 PMC1865890

[21] Lenselink CH , Melchers WJG , Quint WGV , Hoebers AMJ , Hendriks JCM , Massuger LFAG , Bekkers RLM . 2008. Sexual behaviour and HPV infections in 18 to 29 year old women in the pre-vaccine era in the Netherlands. PLoS One 3:e3743. doi:10.1371/journal.pone.0003743 19011683 PMC2581437

[22] Konno R , Tamura S , Dobbelaere K , Yoshikawa H . 2011. Prevalence and type distribution of human papillomavirus in healthy Japanese women aged 20 to 25 years old enrolled in a clinical study. Cancer Sci 102:877–882. doi:10.1111/j.1349-7006.2011.01878.x 21251162 PMC11158349

[23] Adebamowo SN , Ma B , Zella D , Famooto A , Ravel J , Adebamowo C , ACCME Research Group . 2017. Mycoplasma hominis and Mycoplasma genitalium in the vaginal microbiota and persistent high-risk human papillomavirus infection. Front Public Health 5:140. doi:10.3389/fpubh.2017.00140 28695118 PMC5483445

[24] Ortiz AP , Tortolero-Luna G , Romaguera J , Pérez CM , González D , Muñoz C , González L , Marrero E , Suárez E , Palefsky JM , Panicker G , Unger ER . 2018. Seroprevalence of HPV 6, 11, 16 and 18 And correlates of exposure in unvaccinated women aged 16-64 years in Puerto Rico. Papillomavirus Res 5:109–113. doi:10.1016/j.pvr.2018.03.006 29555601 PMC5886958

[25] Roachford Os , Alleyne AT , Kuelbs C , Torralba MG , Nelson KE . 2021. The cervicovaginal microbiome and its resistome in a random selection of Afro-Caribbean women. Human Microbiome Journal 20:100079. doi:10.1016/j.humic.2021.100079

[26] Vargas-Robles D , Morales N , Rodríguez I , Nieves T , Godoy-Vitorino F , Alcaraz LD , Pérez M-E , Ravel J , Forney LJ , Domínguez-Bello MG . 2020. Changes in the vaginal microbiota across a gradient of urbanization. Sci Rep 10:12487. doi:10.1038/s41598-020-69111-x 32719372 PMC7385657

[27] Ling Z , Kong J , Liu F , Zhu H , Chen X , Wang Y , Li L , Nelson KE , Xia Y , Xiang C . 2010. Molecular analysis of the diversity of vaginal microbiota associated with bacterial vaginosis. BMC Genomics 11:1–16. doi:10.1186/1471-2164-11-488 20819230 PMC2996984

[28] van Houdt R , Ma B , Bruisten SM , Speksnijder A , Ravel J , de Vries HJC . 2018. Lactobacillus iners-dominated vaginal microbiota is associated with increased susceptibility to Chlamydia trachomatis infection in Dutch women: a case-control study. Sex Transm Infect 94:117–123. doi:10.1136/sextrans-2017-053133 28947665 PMC6083440

[29] Norenhag J , Du J , Olovsson M , Verstraelen H , Engstrand L , Brusselaers N . 2020. The vaginal microbiota, human papillomavirus and cervical dysplasia: a systematic review and network meta‐analysis. BJOG 127:171–180. doi:10.1111/1471-0528.15854 31237400

[30] Hyman RW , Fukushima M , Jiang H , Fung E , Rand L , Johnson B , Vo KC , Caughey AB , Hilton JF , Davis RW , Giudice LC . 2014. Diversity of the vaginal microbiome correlates with preterm birth. Reprod Sci 21:32–40. doi:10.1177/1933719113488838 23715799 PMC3857766

[31] You Y-A , Park S , Kim K , Kwon EJ , Hur YM , Kim SM , Lee G , Ansari A , Park J , Kim YJ . 2022. Transition in vaginal lactobacillus species during pregnancy and prediction of preterm birth in Korean women. Sci Rep 12:22303. doi:10.1038/s41598-022-26058-5 36566290 PMC9789976

[32] Gajer P , Brotman RM , Bai G , Sakamoto J , Schütte UME , Zhong X , Koenig SSK , Fu L , Ma ZS , Zhou X , Abdo Z , Forney LJ , Ravel J . 2012. Temporal dynamics of the human vaginal microbiota. Sci Transl Med 4:132ra52. doi:10.1126/scitranslmed.3003605 PMC3722878 22553250

[33] Antonio MA , Hawes SE , Hillier SL . 1999. The identification of vaginal lactobacillus species and the demographic and microbiologic characteristics of women colonized by these species. J Infect Dis 180:1950–1956. doi:10.1086/315109 10558952

[34] Mitchell C , Fredricks D , Agnew K , Hitti J . 2015. Hydrogen peroxide-producing lactobacilli are associated with lower levels of vaginal Interleukin-1Beta, independent of bacterial vaginosis. Sex Transm Dis 42:358–363. doi:10.1097/OLQ.0000000000000298 26222747 PMC4520248

[35] Amabebe E , Anumba DOC . 2018. The vaginal microenvironment: the physiologic role of Lactobacilli. Front Med (Lausanne) 5:181. doi:10.3389/fmed.2018.00181 29951482 PMC6008313

[36] Mossop H , Linhares IM , Bongiovanni AM , Ledger WJ , Witkin SS . 2011. Influence of lactic acid on endogenous and viral RNA-induced immune mediator production by vaginal epithelial cells. Obstet Gynecol 118:840–846. doi:10.1097/AOG.0b013e31822da9e9 21934447

[37] Boskey ER , Cone RA , Whaley KJ , Moench TR . 2001. Origins of vaginal acidity: high D/L lactate ratio is consistent with bacteria being the primary source. Hum Reprod 16:1809–1813. doi:10.1093/humrep/16.9.1809 11527880

[38] Anahtar MN , Byrne EH , Doherty KE , Bowman BA , Yamamoto HS , Soumillon M , Padavattan N , Ismail N , Moodley A , Sabatini ME , Ghebremichael MS , Nusbaum C , Huttenhower C , Virgin HW , Ndung’u T , Dong KL , Walker BD , Fichorova RN , Kwon DS . 2015. Cervicovaginal bacteria are a major modulator of host inflammatory responses in the female genital tract. Immunity 42:965–976. doi:10.1016/j.immuni.2015.04.019 25992865 PMC4461369

[39] Castle PE , Hillier SL , Rabe LK , Hildesheim A , Herrero R , Bratti MC , Sherman ME , Burk RD , Rodriguez AC , Alfaro M , Hutchinson ML , Morales J , Schiffman M . 2001. An association of cervical inflammation with high-grade cervical neoplasia in women infected with oncogenic human papillomavirus (HPV). Cancer Epidemiol Biomarkers Prev 10:1021–1027. doi:http://cebp.aacrjournals.org/content/10/10/1021 11588127

[40] Lev-Sagie A , De Seta F , Verstraelen H , Ventolini G , Lonnee-Hoffmann R , Vieira-Baptista P . 2022. The vaginal microbiome: II. vaginal dysbiotic conditions. J Low Genit Tract Dis 26:79–84. doi:10.1097/LGT.0000000000000644 34928257 PMC8719518

[41] Fredricks DN , Fiedler TL , Marrazzo JM . 2005. Molecular identification of bacteria associated with bacterial vaginosis. N Engl J Med 353:1899–1911. doi:10.1056/NEJMoa043802 16267321

[42] Hillier SL , Nugent RP , Eschenbach DA , Krohn MA , Gibbs RS , Martin DH , Cotch MF , Edelman R , Pastorek JG , Rao AV , McNellis D , Regan JA , Carey JC , Klebanoff MA . 1995. Association between bacterial vaginosis and preterm delivery of a low-birth-weight infant. N Engl J Med 333:1737–1742. doi:10.1056/NEJM199512283332604 7491137

[43] Donders G , Van Calsteren K , Bellen G , Reybrouck R , Van den Bosch T , Riphagen I , Van Lierde S . 2009. Predictive value for preterm birth of abnormal vaginal flora, bacterial vaginosis and aerobic vaginitis during the first trimester of pregnancy. BJOG 116:1315–1324. doi:10.1111/j.1471-0528.2009.02237.x 19538417

[44] Sewankambo N , Gray RH , Wawer MJ , Paxton L , McNairn D , Wabwire-Mangen F , Serwadda D , Li C , Kiwanuka N , Hillier SL , Rabe L , Gaydos CA , Quinn TC , Konde-Lule J . 1997. HIV-1 infection associated with abnormal vaginal flora morphology and bacterial vginosis. The Lancet 350:546–550. doi:10.1016/S0140-6736(97)01063-5 9284776

[45] Wahl SM , McNeely TB , Janoff EN , Shugars D , Worley P , Tucker C , Orenstein JM . 1997. Secretory leukocyte protease inhibitor (SLPI) in mucosal fluids inhibits HIV‐1. Oral Dis 3 Suppl 1:S64–S69. doi:10.1111/j.1601-0825.1997.tb00377.x 9456660

[46] Brotman RM , Shardell MD , Gajer P , Fadrosh D , Chang K , Silver MI , Viscidi RP , Burke AE , Ravel J , Gravitt PE . 2014. Association between the vaginal microbiota, menopause status and signs of vulvovaginal atrophy. Menopause 21:450–458. doi:10.1097/GME.0b013e3182a4690b 24080849 PMC3994184

[47] Muhleisen AL , Herbst-Kralovetz MM . 2016. Menopause and the vaginal microbiome. Maturitas 91:42–50. doi:10.1016/j.maturitas.2016.05.015 27451320

[48] Dothard MI , Allard SM , Gilbert JA . 2023. The effects of hormone replacement therapy on the microbiomes of postmenopausal women. Climacteric 26:182–192. doi:10.1080/13697137.2023.2173568 37051868

[49] Freitas AC , Chaban B , Bocking A , Rocco M , Yang S , Hill JE , Money DM , VOGUE Research Group . 2017. The vaginal microbiome of pregnant women is less rich and diverse, with lower prevalence of mollicutes, compared to non-pregnant women. Sci Rep 7:9212. doi:10.1038/s41598-017-07790-9 28835692 PMC5569030

[50] Serrano MG , Parikh HI , Brooks JP , Edwards DJ , Arodz TJ , Edupuganti L , Huang B , Girerd PH , Bokhari YA , Bradley SP , Brooks JL , Dickinson MR , Drake JI , Duckworth RA 3rd , Fong SS , Glascock AL , Jean S , Jimenez NR , Khoury J , Koparde VN , Lara AM , Lee V , Matveyev AV , Milton SH , Mistry SD , Rozycki SK , Sheth NU , Smirnova E , Vivadelli SC , Wijesooriya NR , Xu J , Xu P , Chaffin DO , Sexton AL , Gravett MG , Rubens CE , Hendricks-Muñoz KD , Jefferson KK , Strauss JF 3rd , Fettweis JM , Buck GA . 2019. Racioethnic diversity in the dynamics of the vaginal microbiome during pregnancy. Nat Med 25:1001–1011. doi:10.1038/s41591-019-0465-8 31142850 PMC6746180

[51] Mirmonsef P , Hotton AL , Gilbert D , Burgad D , Landay A , Weber KM , Cohen M , Ravel J , Spear GT . 2014. Free glycogen in vaginal fluids is associated with Lactobacillus colonization and low vaginal pH. PLoS One 9:e102467. doi:10.1371/journal.pone.0102467 25033265 PMC4102502

[52] Cruickshank R . 1934. The conversion of the glycogen of the vagina into lactic acid. J Pathol 39:213–219. doi:10.1002/path.1700390118

[53] O’Hanlon DE , Moench TR , Cone RA . 2013. Vaginal pH and microbicidal lactic acid when lactobacilli dominate the microbiota. PLoS One 8:e80074. doi:10.1371/journal.pone.0080074 24223212 PMC3819307

[54] Amir M , Brown JA , Rager SL , Sanidad KZ , Ananthanarayanan A , Zeng MY . 2020. Maternal microbiome and infections in pregnancy. Microorganisms 8:12. doi:10.3390/microorganisms8121996 PMC7765218 33333813

[55] Shannon B , Yi TJ , Perusini S , Gajer P , Ma B , Humphrys MS , Thomas-Pavanel J , Chieza L , Janakiram P , Saunders M , Tharao W , Huibner S , Shahabi K , Ravel J , Rebbapragada A , Kaul R . 2017. Association of HPV infection and clearance with cervicovaginal immunology and the vaginal microbiota. Mucosal Immunol 10:1310–1319. doi:10.1038/mi.2016.129 28120845 PMC5526752

[56] Tuominen H , Rautava S , Syrjänen S , Collado MC , Rautava J . 2018. HPV infection and bacterial microbiota in the placenta, uterine cervix and oral mucosa. Sci Rep 8:9787. doi:10.1038/s41598-018-27980-3 29955075 PMC6023934

[57] Di Paola M , Sani C , Clemente AM , Iossa A , Perissi E , Castronovo G , Tanturli M , Rivero D , Cozzolino F , Cavalieri D , Carozzi F , De Filippo C , Torcia MG . 2017. Characterization of cervico-vaginal microbiota in women developing persistent high-risk human papillomavirus infection. Sci Rep 7:10200. doi:10.1038/s41598-017-09842-6 28860468 PMC5579045

[58] Qingqing B , Jie Z , Songben Q , Juan C , Lei Z , Mu X . 2021. Cervicovaginal microbiota dysbiosis correlates with HPV persistent infection. Microb Pathog 152:104617. doi:10.1016/j.micpath.2020.104617 33207260

[59] Kovachev SM . 2020. Cervical cancer and vaginal microbiota changes. Arch Microbiol 202:323–327. doi:10.1007/s00203-019-01747-4 31659380

[60] Schwabe RF , Jobin C . 2013. The microbiome and cancer. Nat Rev Cancer 13:800–812. doi:10.1038/nrc3610 24132111 PMC3986062

[61] Chen Y , Qiu X , Wang W , Li D , Wu A , Hong Z , Di W , Qiu L . 2020. Human papillomavirus infection and cervical intraepithelial neoplasia progression are associated with increased vaginal microbiome diversity in a Chinese cohort. BMC Infect Dis 20:1–12. doi:10.1186/s12879-020-05324-9 PMC7449047 32842982

[62] Mitra A , MacIntyre DA , Lee YS , Smith A , Marchesi JR , Lehne B , Bhatia R , Lyons D , Paraskevaidis E , Li JV , Holmes E , Nicholson JK , Bennett PR , Kyrgiou M . 2015. Cervical intraepithelial neoplasia disease progression is associated with increased vaginal microbiome diversity. Sci Rep 5:16865. doi:10.1038/srep16865 26574055 PMC4648063

[63] Orlando PA , Gatenby RA , Giuliano AR , Brown JS . 2012. Evolutionary ecology of human papillomavirus: trade-offs, coexistence, and origins of high-risk and low-risk types. J Infect Dis 205:272–279. doi:10.1093/infdis/jir717 22090448 PMC3244363

[64] Luostarinen T , af Geijersstam V , Bjørge T , Eklund C , Hakama M , Hakulinen T , Jellum E , Koskela P , Paavonen J , Pukkala E , Schiller JT , Thoresen S , Youngman LD , Dillner J , Lehtinen M . 1999. No excess risk of cervical carcinoma among women seropositive for both HPV16 and HPV6/11. Int J Cancer 80:818–822. doi:10.1002/(sici)1097-0215(19990315)80:6<818::aid-ijc4>3.0.co;2-t 10074912

[65] Sundström K , Ploner A , Arnheim-Dahlström L , Eloranta S , Palmgren J , Adami H-O , Ylitalo Helm N , Sparén P , Dillner J . 2015. Interactions between high-and low-risk HPV types reduce the risk of squamous cervical cancer. J Natl Cancer Inst 107:djv185. doi:10.1093/jnci/djv185 26160881 PMC5964715

[66] Silins I , Wang Z , Åvall-Lundqvist E , Frankendal B , Vikmanis U , Sapp M , Schiller JT , Dillner J . 1999. Serological evidence for protection by human papillomavirus (HPV) type 6 infection against HPV type 16 cervical carcinogenesis. J Gen Virol 80 (Pt 11):2931–2936. doi:10.1099/0022-1317-80-11-2931 10580926

[67] Brooks JP , Buck GA , Chen G , Diao L , Edwards DJ , Fettweis JM , Huzurbazar S , Rakitin A , Satten GA , Smirnova E , Waks Z , Wright ML , Yanover C , Zhou Y-H . 2017. Changes in vaginal community state types reflect major shifts in the microbiome. Microb Ecol Health Dis 28:1303265. doi:10.1080/16512235.2017.1303265 28572753 PMC5443090

[68] Ravel J , Brotman RM , Gajer P , Ma B , Nandy M , Fadrosh DW , Sakamoto J , Koenig SS , Fu L , Zhou X , Hickey RJ , Schwebke JR , Forney LJ . 2013. Daily temporal dynamics of vaginal microbiota before, during and after episodes of bacterial vaginosis. Microbiome 1:1–6. doi:10.1186/2049-2618-1-29 24451163 PMC3968321

[69] McInnes P , Cutting M . 2010. Manual of procedures – human microbiome project: core microbiome sampling, protocol A. HMP Protocol # 07-001. Available from: https://www.ncbi.nlm.nih.gov/projects/gap/cgi-bin/GetPdf.cgi?id=phd003190.2

[70] Huang Y-E , Wang Y , He Y , Ji Y , Wang L-P , Sheng H-F , Zhang M , Huang Q-T , Zhang D-J , Wu J-J , Zhong M , Zhou H-W . 2015. Homogeneity of the vaginal microbiome at the cervix, posterior fornix, and vaginal canal in pregnant Chinese women. Microb Ecol 69:407–414. doi:10.1007/s00248-014-0487-1 25230887

[71] Caporaso JG , Lauber CL , Walters WA , Berg-Lyons D , Huntley J , Fierer N , Owens SM , Betley J , Fraser L , Bauer M , Gormley N , Gilbert JA , Smith G , Knight R . 2012. Ultra-high-throughput microbial community analysis on the Illumina HiSeq and MiSeq platforms. ISME J 6:1621–1624. doi:10.1038/ismej.2012.8 22402401 PMC3400413

[72] Chorna N , Romaguera J , Godoy-Vitorino F . 2020. Cervicovaginal microbiome and urine metabolome paired analysis reveals niche partitioning of the microbiota in patients with human papilloma virus infections. Metabolites 10:36. doi:10.3390/metabo10010036 31952112 PMC7022855

[73] Derilus D , Godoy-Vitorino F , Rosado H , Agosto E , Dominguez-Bello MG , Cavallin H . 2020. An in-depth survey of the microbial landscape of the walls of a neonatal operating room. PLoS One 15:e0230957. doi:10.1371/journal.pone.0230957 32243474 PMC7122808

[74] Rodríguez-Barreras R , Tosado-Rodríguez EL , Godoy-Vitorino F . 2021. Trophic niches reflect compositional differences in microbiota among caribbean sea urchins. PeerJ 9:e12084. doi:10.7717/peerj.12084 34540373 PMC8415288

[75] Gonzalez A , Navas-Molina JA , Kosciolek T , McDonald D , Vázquez-Baeza Y , Ackermann G , DeReus J , Janssen S , Swafford AD , Orchanian SB , Sanders JG , Shorenstein J , Holste H , Petrus S , Robbins-Pianka A , Brislawn CJ , Wang M , Rideout JR , Bolyen E , Dillon M , Caporaso JG , Dorrestein PC , Knight R . 2018. Qiita: rapid, web-enabled microbiome meta-analysis. Nat Methods 15:796–798. doi:10.1038/s41592-018-0141-9 30275573 PMC6235622

[76] Crothers BA . 2005. The bethesda system 2001: update on terminology and application. Clin Obstet Gynecol 48:98–107. doi:10.1097/01.grf.0000151572.99437.a5 15725862

[77] Bokulich NA , Kaehler BD , Rideout JR , Dillon M , Bolyen E , Knight R , Huttley GA , Gregory Caporaso J . 2018. Optimizing taxonomic classification of marker-gene amplicon sequences with QIIME 2’s Q2-feature-classifier plugin. Microbiome 6:90. doi:10.1186/s40168-018-0470-z 29773078 PMC5956843

[78] NIH HMP Working Group, Peterson J , Garges S , Giovanni M , McInnes P , Wang L , Schloss JA , Bonazzi V , McEwen JE , Wetterstrand KA , Deal C , Baker CC , Di Francesco V , Howcroft TK , Karp RW , Lunsford RD , Wellington CR , Belachew T , Wright M , Giblin C , David H , Mills M , Salomon R , Mullins C , Akolkar B , Begg L , Davis C , Grandison L , Humble M , Khalsa J , Little AR , Peavy H , Pontzer C , Portnoy M , Sayre MH , Starke-Reed P , Zakhari S , Read J , Watson B , Guyer M . 2009. The NIH human microbiome project. Genome Res 19:2317–2323. doi:10.1101/gr.096651.109 19819907 PMC2792171

[79] Usyk M , Zolnik CP , Castle PE , Porras C , Herrero R , Gradissimo A , Gonzalez P , Safaeian M , Schiffman M , Burk RD , Costa Rica HPV Vaccine Trial (CVT) Group . 2020. Cervicovaginal microbiome and natural history of HPV in a longitudinal study. PLoS Pathog 16:e1008376. doi:10.1371/journal.ppat.1008376 32214382 PMC7098574

[80] Usyk M , Schlecht NF , Pickering S , Williams L , Sollecito CC , Gradissimo A , Porras C , Safaeian M , Pinto L , Herrero R , Strickler HD , Viswanathan S , Nucci-Sack A , Diaz A , Costa Rica HPV Vaccine Trial (CVT) Group, Burk RD . 2022. molBV reveals immune landscape of bacterial vaginosis and predicts human papillomavirus infection natural history. Nat Commun 13:233. doi:10.1038/s41467-021-27628-3 35017496 PMC8752746

[81] Van Der Pol WJ , Kumar R , Morrow CD , Blanchard EE , Taylor CM , Martin DH , Lefkowitz EJ , Muzny CA . 2019. In Silico and experimental evaluation of primer sets for species-level resolution of the vaginal Microbiota using 16S Ribosomal RNA gene sequencing. J Infect Dis 219:305–314. doi:10.1093/infdis/jiy508 30535155 PMC6941453

[82] Anderson MJ . 2014. Permutational multivariate analysis of variance (PERMANOVA), p 1–15. In Wiley statsref: statistics reference online

[83] Toikka A , Willberg E , Mäkinen V , Toivonen T , Oksanen J . 2020. Vegan: community ecology package. doi:10.1016/j.dib.2020.105601 PMC7200931 32382610

[84] Simpson GL . 2016. Permute: functions for generating restricted permutations of data. R package version 0.9-4. URL CRAN. R-Project. Org/Package= Permute

[85] Mallick H , Rahnavard A , McIver LJ , Ma S , Zhang Y , Nguyen LH , Tickle TL , Weingart G , Ren B , Schwager EH , Chatterjee S , Thompson KN , Wilkinson JE , Subramanian A , Lu Y , Waldron L , Paulson JN , Franzosa EA , Bravo HC , Huttenhower C . 2021. Multivariable association discovery in population-scale meta-omics studies. PLoS Comput Biol 17:e1009442. doi:10.1371/journal.pcbi.1009442 34784344 PMC8714082

[86] Linz AM , Crary BC , Shade A , Owens S , Gilbert JA , Knight R , McMahon KD . 2017. Bacterial community composition and dynamics spanning five years in freshwater bog lakes mSphere 2:e00296-17. doi:10.1128/mSphere.00296-17 28680968 PMC5489657

[87] Kuznetsova A , Brockhoff PB , Christensen RHB . 2017. lmerTest package: tests in linear mixed effects models. J Stat Soft 82:1–26. doi:10.18637/jss.v082.i13

[88] Lenth R . 2019. Package 'emmeans' doi:10.1080/00031305.1980.10483031

[89] Signorell A . 2022. Desctools: tools for descriptive statistics. R package

