# Supplementary figures and images for: The cervical microbiota of Hispanics living in Puerto Rico is nonoptimal regardless of HPV status
Source: mSystems. 2023 Aug 3;8(4):e00357-23. doi: 10.1128/msystems.00357-23 (PMC10469956; doi:10.1128/msystems.00357-23)

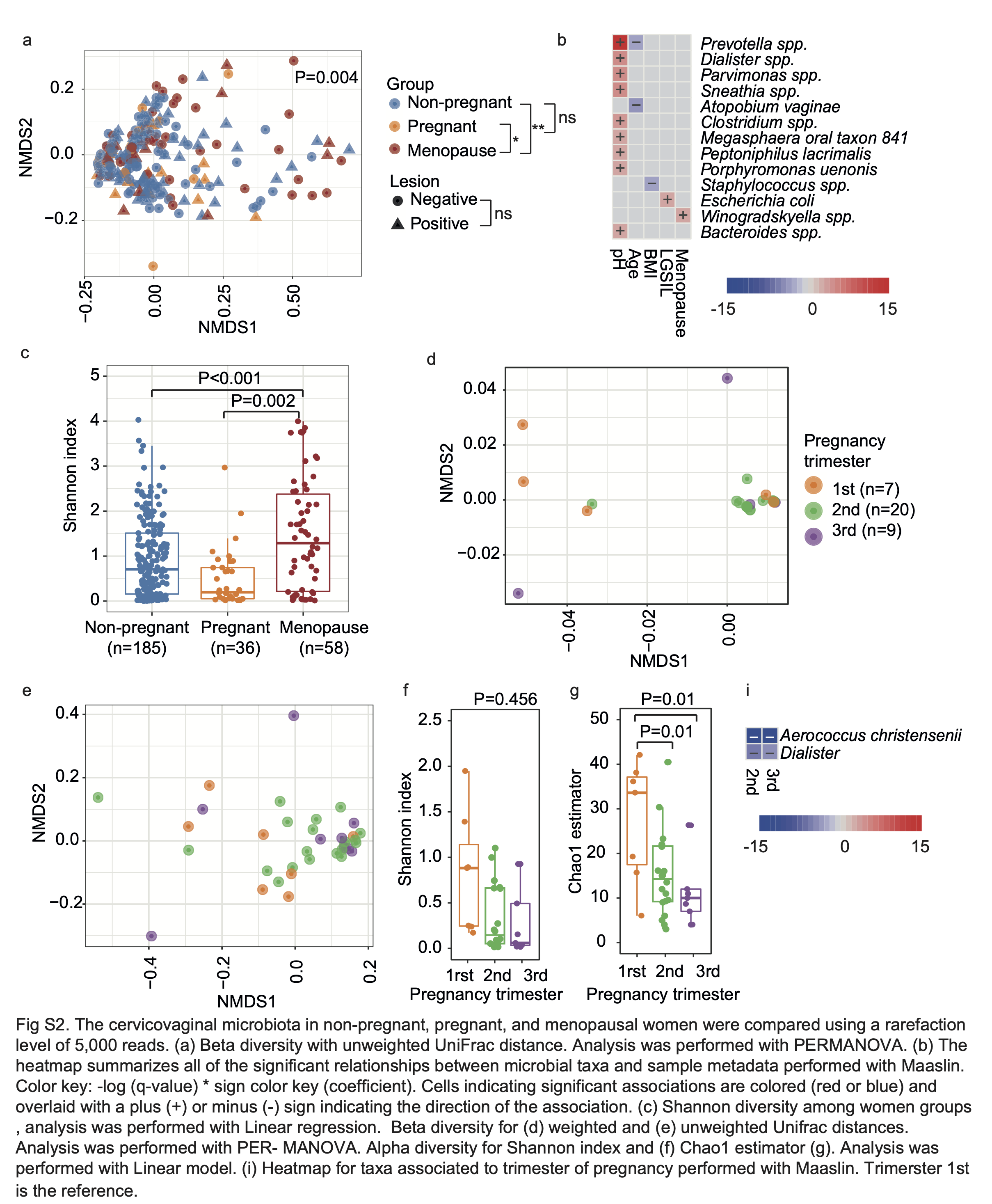

Supplement: Fig. S2 — The cervicovaginal microbiota in nonpregnant, pregnant, and menopausal women. [file msystems.00357-23-s0002.tif]

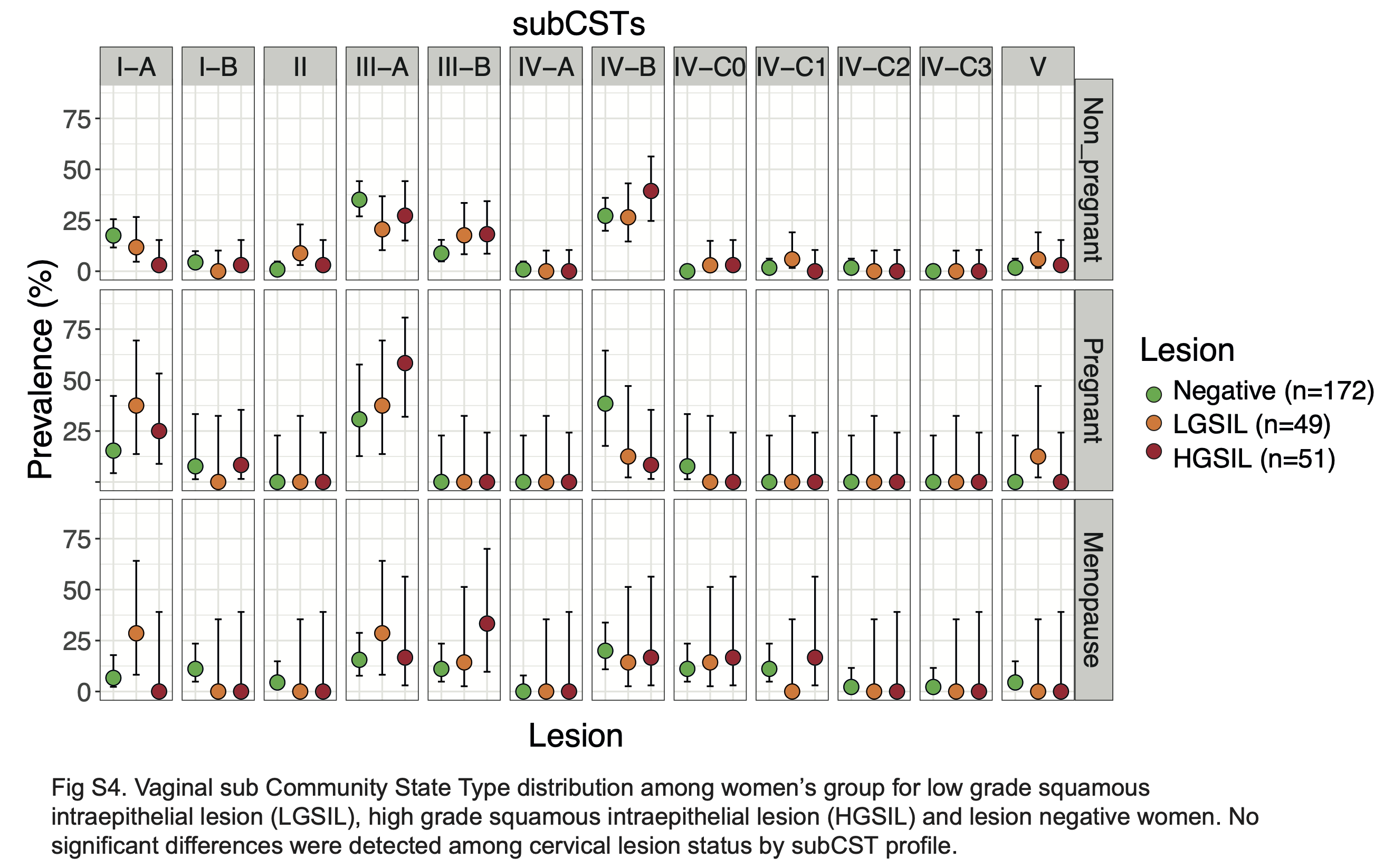

Supplement: Fig. S4 — Vaginal sub-Community State type distribution among women's group for low-grade squamous intraepithelial lesion (LGSIL), high-grade squamous intraepithelial lesions (HGSIL), and women negative for cervical lesions. [file msystems.00357-23-s0004.tif]

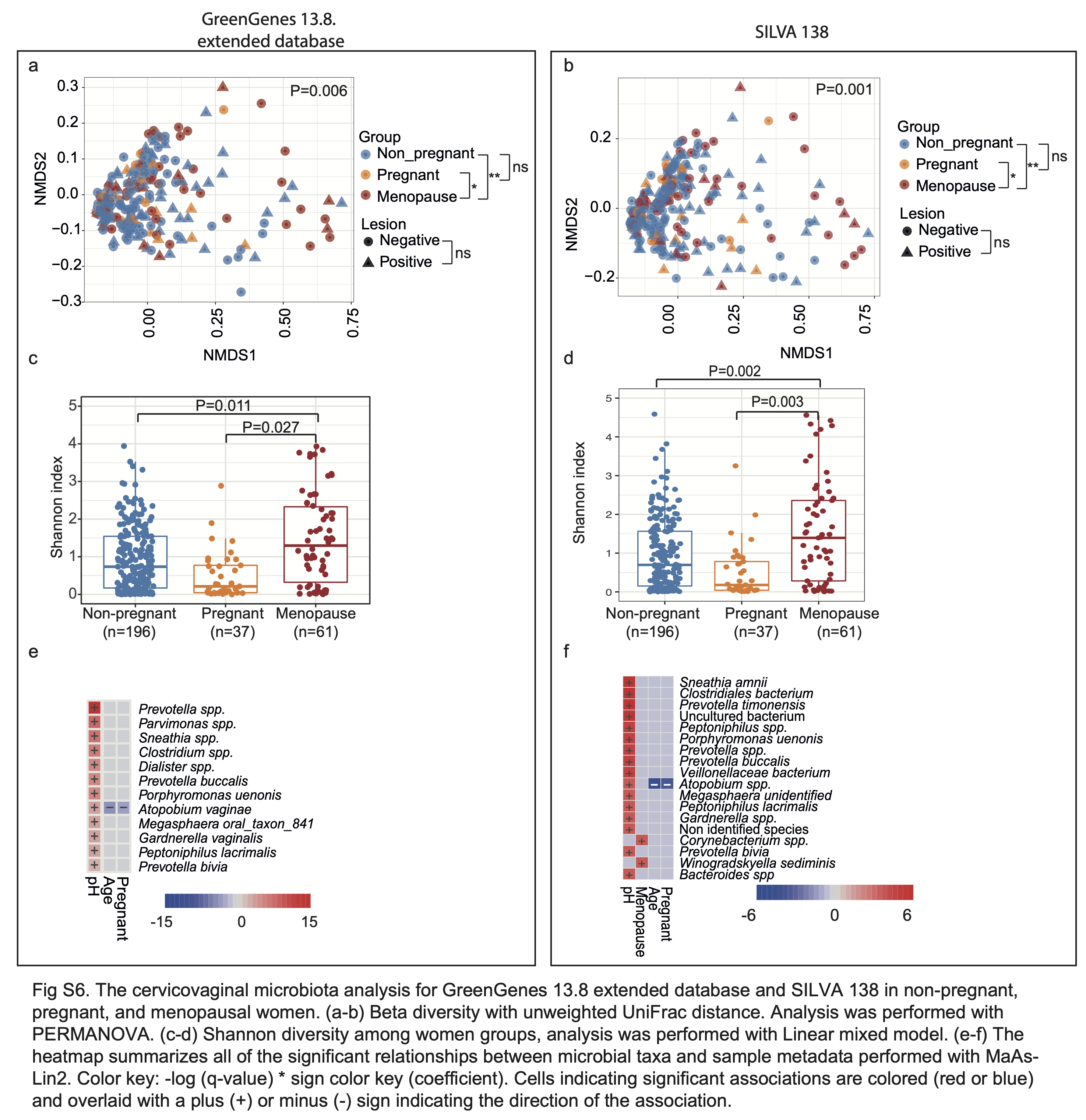

Supplement: Fig. S6 — The cervicovaginal microbiota analysis for GreenGenes 138 extended database and SILVA 138 in nonpregnant, pregnant, and menopausal women. [file msystems.00357-23-s0006.tif]

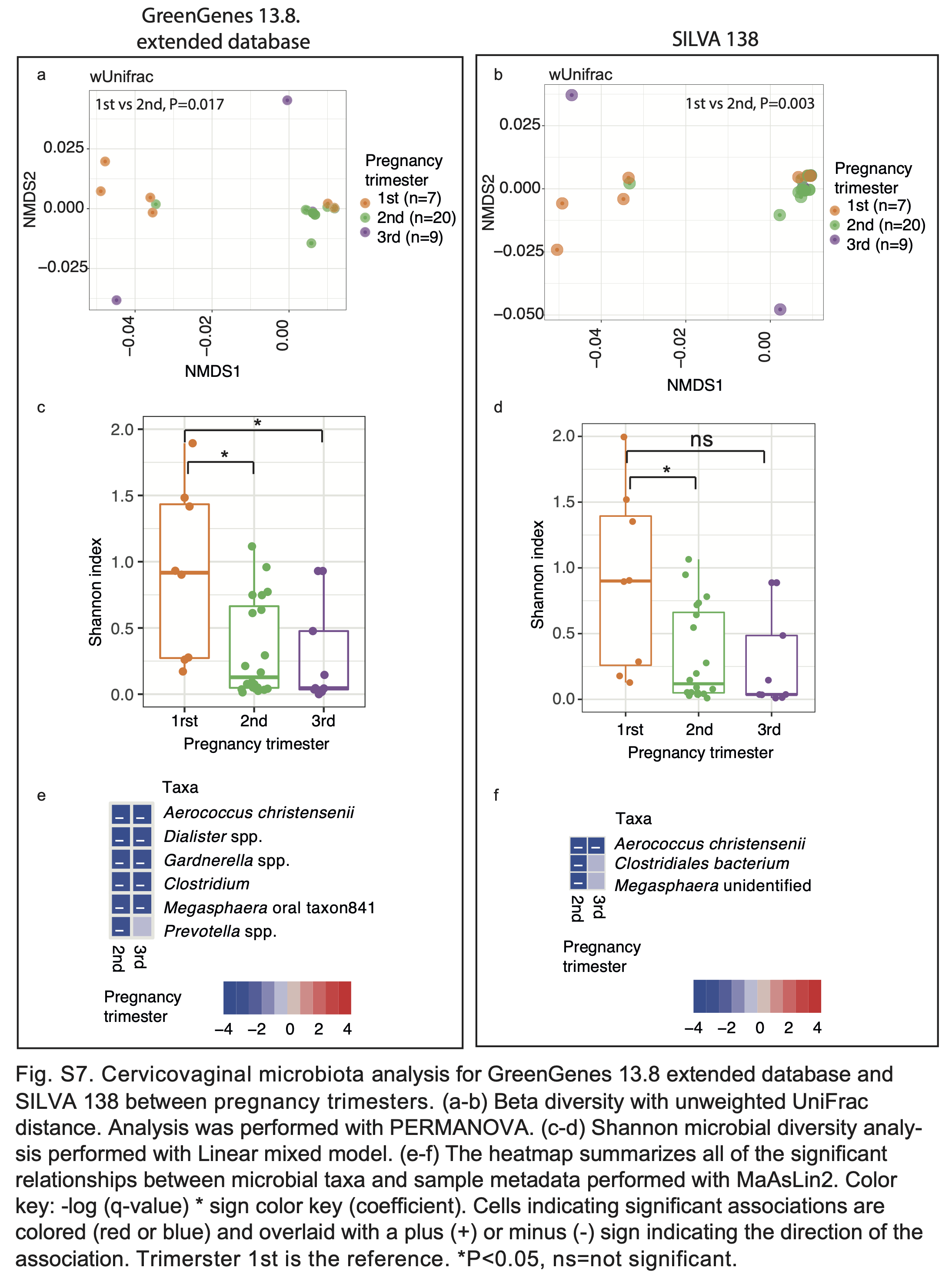

Supplement: Fig. S7 — Cervicovaginal microbiota analysis for GreenGenes 13.8 extended database and SILVA 138 between pregnancy trimesters. [file msystems.00357-23-s0007.tif]
