# Supplementary material for: The cervical microbiota of Hispanics living in Puerto Rico is nonoptimal regardless of HPV status
Source: mSystems. 2023 Aug 3;8(4):e00357-23. doi: 10.1128/msystems.00357-23 (PMC10469956; doi:10.1128/msystems.00357-23)
Supplement: Supplemental tables — Tables S1 to S8. [file msystems.00357-23-s0009.pdf]

Table S1. Summary of sequences and ASV before and after rarefaction.

	Parameters	Not rarefied	Rarefied
Sequences	N samples	337	333
	Sum seqs	8,865,727	590,409
	Mean seqs	26,308	1,773
	Sd seqs	20,059	-
	Max seqs/sample	80,253	1,773
	Min seqs/sample	187	1,773
ASVs	Sum ASVs	10,262	6,581
	Mean ASVs	59	45
	Sd ASVs	90	70
	Max ASVs/sample	720	527
	Min ASVs/sample	4	2
Species	Sum Species	1,023	805
	Mean Species	30	21
	Sd Species	35	28
	Max Species/sample	249	188
	Min Species/sample	1	1

Table S2. Prevalence of HPV genotypes among women groups.

HPV type	Non pregnant (N=151)	Pregnant (N=27)	Menopause (N=42)	TOTAL (N=220)	*P value &*
**HR51**	44 (29.1%)	8 (29.6%)	17 (40.5%)	69 (31.4%)	*0.378*
**HR16**	33 (21.9%)	5 (18.5%)	8 (19.0%)	46 (20.9%)	*0.903*
**HR33**	24 (15.9%)	5 (18.5%)	8 (19.0%)	37 (16.8%)	*0.818*
**HR52**	21 (13.9%)	7 (25.9%)	4 (9.5%)	32 (14.5%)	*0.159*
**HR56**	24 (15.9%)	3 (11.1%)	5 (11.9%)	32 (14.5%)	*0.798*
**HR66**	22 (14.6%)	0 (0%)	7 (16.7%)	29 (13.2%)	*0.060*
**HR45**	14 (9.3%)	1 (3.7%)	1 (2.4%)	16 (7.3%)	*0.313*
**HR35**	10 (6.6%)	2 (7.4%)	3 (7.1%)	15 (6.8%)	*1.000*
**HR68**	8 (5.3%)	2 (7.4%)	5 (11.9%)	15 (6.8%)	*0.236*
**HR39**	9 (6.0%)	1 (3.7%)	2 (4.8%)	12 (5.5%)	*1.000*
**HR58**	6 (4.0%)	1 (3.7%)	4 (9.5%)	11 (5.0%)	*0.338*
**HR59**	3 (2.0%)	0 (0%)	5 (11.9%)	8 (3.6%)	*0.024 (Padj=0.272)*
**HR31**	1 (0.7%)	0 (0%)	4 (9.5%)	5 (2.3%)	*0.008 (Padj=0.173)*
**HR18**	2 (1.3%)	0 (0%)	0 (0%)	2 (0.9%)	*1.000*
**LR53**	39 (25.8%)	3 (11.1%)	8 (19.0%)	50 (22.7%)	*0.217*
**LR74**	16 (10.6%)	3 (11.1%)	3 (7.1%)	22 (10.0%)	*0.831*
**LR44**	8 (5.3%)	0 (0%)	1 (2.4%)	9 (4.1%)	*0.659*
**LR6**	7 (4.6%)	0 (0%)	1 (2.4%)	8 (3.6%)	*0.740*
**LR70**	5 (3.3%)	0 (0%)	1 (2.4%)	6 (2.7%)	*1.000*
**LR54**	3 (2.0%)	0 (0%)	1 (2.4%)	4 (1.8%)	*1.000*
**LR40**	2 (1.3%)	0 (0%)	0 (0%)	2 (0.9%)	*1.000*
**LR19**	1 (0.7%)	0 (0%)	0 (0%)	1 (0.5%)	*1.000*
**LR43**	1 (0.7%)	0 (0%)	0 (0%)	1 (0.5%)	*1.000*

& Fisher's Exact Test. P value adjustment with "False Discovery Rate" method

Table S3. Maaslin detailed results. It shows each of the taxa is significantly associated with a particular variable. It shows the model coefficient, the standard error, the number of samples, the number of samples with at least one sequence for a particular taxon, the P-value, and the adjusted P-value.

**Feature**	**metadata**	**value**	**coef**	**stderr**	**N**	**N.not.0**	***P-value &***	***P adj. &***
Prevotella	pH	pH	1.528	0.230	294	115	*4.8E-10*	*1.8E-07*
Parvimonas	pH	pH	0.671	0.133	294	41	*1.3E-06*	*2.3E-04*
Sneathia	pH	pH	1.283	0.275	294	89	*6.4E-06*	*7.9E-04*
Clostridium	pH	pH	0.975	0.214	294	67	*1.1E-05*	*1.0E-03*
Dialister	pH	pH	0.550	0.130	294	53	*3.9E-05*	*2.7E-03*
Prevotella buccalis	pH	pH	0.857	0.204	294	58	*4.4E-05*	*2.7E-03*
Porphyromonas uenonis	pH	pH	0.540	0.137	294	30	*1.2E-04*	*6.3E-03*
Atopobium vaginae	Age	Age	-1.378	0.367	294	106	*2.4E-04*	*1.1E-02*
Megasphaera oral taxon 841	pH	pH	0.860	0.248	294	73	*7.0E-04*	*2.9E-02*
Peptoniphilus lacrimalis	pH	pH	0.353	0.105	294	32	*9.6E-04*	*3.3E-02*
Gardnerella vaginalis	pH	pH	1.201	0.358	294	166	*1.0E-03*	*3.3E-02*
Atopobium vaginae	Group	Pregnant	-3.017	0.904	294	106	*1.1E-03*	*3.3E-02*
Atopobium vaginae	pH	pH	0.884	0.270	294	106	*1.3E-03*	*3.8E-02*
Prevotella bivia	pH	pH	0.630	0.201	294	68	*2.0E-03*	*5.0E-02*

& Fisher's Exact Test. P value adjustment with "False Discovery Rate" method

Table S4. Distribution of CST on cervical lesions status, only among non-pregnant women

	Cervical Lesion &&			
	**Negative (N=112)**	**Positive (N=69)**	**TOTAL (N=196)**	***P value &&***
**CST1**				*0.039*
I	25 (22.3%)	6 (8.7%)	34 (17.3%)	
II	1 (0.9%)	4 (5.8%)	6 (3.1%)	
III	48 (42.9%)	30 (43.5%)	83 (42.3%)	
IV	36 (32.1%)	26 (37.7%)	68 (34.7%)	
V	2 (1.8%)	3 (4.3%)	5 (2.6%)	

& Positive samples includes the HGSIL or LGSIL

&& Fisher's Exact Test

Table S5. Distribution of CST on cervical HPV by HPV risk types, only among non-pregnant women

	Cervical HPV type by risk				
	**Negative (N=44)**	**Only_low_risk (N=17)**	**High_Risk (N=133)**	**TOTAL (N=196)**	***P value &***
**CST1**					*0.039*
I	9 (20.5%)	4 (23.5%)	21 (15.8%)	34 (17.3%)	
II	0 (0%)	2 (11.8%)	4 (3.0%)	6 (3.1%)	
III	15 (34.1%)	7 (41.2%)	59 (44.4%)	83 (42.3%)	
IV	19 (43.2%)	2 (11.8%)	47 (35.3%)	68 (34.7%)	
V	1 (2.3%)	2 (11.8%)	2 (1.5%)	5 (2.6%)	

& Fisher's Exact Test

Table S6. Comparison of Hispanic women CSTs between Puerto Rico and the USA

	Prevalence among Hispanic (%)		
**CST**	**PR (N=196)**	**USA (N=97)**	***P value &***
I	17.3	14.4	*0.616*
II	3.1	7.2	*0.132*
III	42.3	36.1	*0.315*
IV	34.7	38.1	*0.881*
V	2.6	4.1	*0.485*

& Fisher's Exact Test

Table S7. Changes of cervical lesion diagnosis in relationship with changes in the CST in 2 visits from 4 to 16 months apart.

	Visit1-Visit2				
**Variable**	**lac_dom-lac_dom**	**lac_dom-div**	**div-lac_dom**	**div-div**	***P value &***
**Cervical lesions**					*0.991*
HGSIL_HGSIL	2	0	0	0	
HGSIL_LGSIL	1	1	0	0	
HGSIL_Negative	3	1	1	3	
LGSIL_LGSIL	2	0	0	0	
LGSIL_Negative	3	1	1	1	
Negative_LGSIL	1	0	1	0	
Negative_Negative	7	2	2	5	

& Fisher's Exact Test

Table S8. Changes of HPV status (any HPV type) diagnosis in relationship with changes in the CST in 2 visits from 4 to 16 months apart.

	Visit1-Visit2				
**Any HPV type**	**lac dom-lac dom**	**lac dom-div**	**div-lac dom**	**div-div**	***P value &***
Negative-Negative	0	1	1	1	*0.225*
Negative-Positive	2	0	1	0	
Positive-Negative	4	0	1	4	
Positive-Positive	13	3	2	4	

& Fisher's Exact Test
